# A conserved molecular logic for neurogenesis to gliogenesis switch in the cerebral cortex

**DOI:** 10.1073/pnas.2321711121

**Published:** 2024-05-07

**Authors:** Xiaoyi G. Liang, Kendy Hoang, Brandon L. Meyerink, Pratiksha Kc, Kitt Paraiso, Li Wang, Ian R. Jones, Yue Zhang, Sol Katzman, Thomas S. Finn, Jeremiah Tsyporin, Fangyuan Qu, Zhaoxu Chen, Axel Visel, Arnold Kriegstein, Yin Shen, Louis-Jan Pilaz, Bin Chen

**Affiliations:** ^a^Department of Molecular, Cell, and Developmental Biology, University of California, Santa Cruz, CA 95064; ^b^Division of Pediatrics and Rare Diseases Group, Sanford Research, Sioux Falls, SD 57104; ^c^Department of Basic Biomedical Sciences, University of South Dakota Sanford School of Medicine, Sioux Falls, SD 57105; ^d^Environmental Genomics & System Biology Division, Lawrence Berkeley National Laboratory, Berkeley, CA 94720; ^e^Eli and Edythe Broad Center of Regeneration Medicine and Stem Cell Research, University of California, San Francisco, CA 94143; ^f^Department of Neurology, University of California, San Francisco, CA 94143; ^g^Institute for Human Genetics, University of California, San Francisco, CA 94143; ^h^Genome Institute, University of California, Santa Cruz, CA 95064; ^i^U.S. Department of Energy Joint Genome Institute, Berkeley, CA 94720; ^j^Department of Molecular and Cell Biology, School of Natural Sciences, University of California, Merced, CA 95343

**Keywords:** gliogenesis, neurogenesis, lineage switch, enhancer, Olig2

## Abstract

During development, neural stem cells in the cerebral cortex, also known as radial glial cells (RGCs), generate excitatory neurons, followed by production of cortical macroglia and inhibitory neurons that migrate to the olfactory bulb (OB). Understanding the mechanisms for this lineage switch is fundamental for unraveling how proper numbers of diverse neuronal and glial cell types are controlled. We and others recently showed that Sonic Hedgehog (Shh) signaling promotes the cortical RGC lineage switch to generate cortical oligodendrocytes and OB interneurons. During this process, cortical RGCs generate intermediate progenitor cells that express critical gliogenesis genes *Ascl1*, *Egfr,* and *Olig2*. The increased *Ascl1* expression and appearance of Egfr^+^ and Olig2^+^ cortical progenitors are concurrent with the switch from excitatory neurogenesis to gliogenesis and OB interneuron neurogenesis in the cortex. While Shh signaling promotes *Olig2* expression in the developing spinal cord, the exact mechanism for this transcriptional regulation is not known. Furthermore, the transcriptional regulation of *Olig2* and *Egfr* has not been explored. Here, we show that in cortical progenitor cells, multiple regulatory programs, including Pax6 and Gli3, prevent precocious expression of *Olig2*, a gene essential for production of cortical oligodendrocytes and astrocytes. We identify multiple enhancers that control *Olig2* expression in cortical progenitors and show that the mechanisms for regulating *Olig2* expression are conserved between the mouse and human. Our study reveals evolutionarily conserved regulatory logic controlling the lineage switch of cortical neural stem cells.

## Significance

In the developing cerebral cortex, neural stem cells switch from generating cortical excitatory neurons to producing cortical glia and olfactory bulb interneurons. This lineage switch is essential for generating appropriate numbers of neuronal and glial cell types in the cortex and requires the transcription of *Olig2* in cortical progenitors. In this study, we describe the identification of multiple enhancers that control the expression of *Olig2* in cortical progenitors using ChIP-seq, CUT&RUN, ATAC-seq, enhancer reporter, and deletion mice. Our study reveals a conserved mechanism for *Olig2* gene expression and neural stem cell lineage regulation between the mouse and human.

The production of proper numbers of diverse neurons and macroglia by neural stem cells (NSCs) is essential for neural circuit formation and brain function. During development, NSCs in the cerebral cortex, known as radial glial cells (RGCs), generate glutamatergic neurons that populate different cortical layers (1). As the generation of excitatory neurons ceases, cortical RGCs switch lineages and generate oligodendrocytes, astrocytes, and GABAergic olfactory bulb (OB) interneurons (2–6). Proper control of this lineage switch ensures the production of diverse neuronal and glial cell types in correct numbers.

The lineages of cortical RGCs have been under extensive scrutiny. Results from cell transplant studies using ferret and rat models suggested that early cortical progenitors are multipotent and sequentially generate diverse excitatory neuron subtypes that populate the cortical layers in an inside-out pattern (1). These results were supported by clonal analysis of individually cultured cortical progenitors (7). A later study reported that early (embryonic day 10.5, or E10.5) Cux2-expressing (Cux2^+^) cortical RGCs were intrinsically lineage-restricted to generate later-born corticocortical projection neurons (8). However, through in vivo lineage analyses of individual RGCs, it was found that early RGCs, including the Cux2^+^ RGCs, are multipotent, and they sequentially generate diverse subtypes of cortical excitatory neurons, followed by OB interneurons and macroglia (2, 9–11).

How is the lineage progression of cortical RGCs regulated to ensure distinct neuronal and glial progenies are generated at different times? By labeling cortical RGCs at the end of excitatory neuron production in mice and performing single-cell RNA-seq analysis of the labeled progeny, Li et al. reported that some late cortical RGCs become translocating RGCs and migrate away from the VZ into the cortical plate to produce astrocytes (12). Other late RGCs remain at the VZ. They divide and generate transient multipotent intermediate progenitors (MIPCs) that are marked by the coexpression of Ascl1, Egfr, and Olig2. These Ascl1^+^Egfr^+^Olig2^+^ MIPCs further divide and generate the progenitors for cortical astrocytes, oligodendrocytes, and inhibitory interneurons that migrate to the OB (4, 12). Immunohistochemical analysis of embryonic mouse and fetal human brains confirms the presence of Ascl1^+^Egfr^+^Olig2^+^ MIPCs in cortical ventricular and subventricular zones (V/SVZ), which give rise to intermediate progenitors for both cortical macroglia and the OB interneurons (4, 12, 13).

Expression of Ascl1, Egfr, and Olig2 is critical for the lineage switch of cortical RGCs. Ascl1 has been reported to be essential for gliogenesis in both the spinal cord and telencephalon (14–16). It is expressed in cortical progenitors from the beginning of cortical neurogenesis (17). Recent elegant studies have shown that Egfr critically regulates cortical gliogenesis in a region-specific pattern (5, 6). During development, it is initially expressed in ventral forebrain progenitors, but its expression is turned on in cortical progenitors around E16.5 in mice, coincident with the onset of cortical RGC lineage switch (12).

*Olig2* encodes a basic helix–loop–helix (bHLH) transcription factor that is required for the specification of oligodendrocyte precursor cells (OPCs) and differentiation of oligodendrocytes (18–21). Previous studies have shown that *Olig2* is also expressed in developing astrocytes and plays a pivotal role in their development (21). Among the forebrain progenitors, *Olig2* is initially expressed in the NSCs located in the medial ganglionic eminence. At E16.5, when the production of excitatory neurons ceases, *Olig2* expression starts to be detected in cortical progenitor cells in the subventricular zone (SVZ), and this expression persists into early postnatal stages (12). Intersectional lineage analysis indicated that in addition to the oligodendrocytes, the Olig2^+^ cortical progenitors give rise to almost all cortical astrocytes and some OB interneurons (12). Indeed, cortex-specific deletion of *Olig2* leads to defective production of both cortical oligodendrocytes and astrocytes (21). However, the molecular mechanism underlying the onset of *Olig2* expression in cortical progenitors has not been determined.

We and others reported that Shh signaling is both necessary and sufficient to promote cortical RGCs to generate OB interneurons and oligodendrocytes (4, 22). We found that Shh signaling activates this lineage switch by causing the degradation of the transcription repressor Gli3 (4). Here, we report that Gli3 and Pax6, transcription factors highly expressed in the cortical RGCs, prevent precocious expression of *Olig2* and the lineage switch of cortical RGCs. We identify multiple *cis*-regulatory sequences that are recruited to the *Olig2* promoter and promote *Olig2* transcription in cortical progenitor cells. We show that these regulatory sequences are conserved in the human genome and are recruited to the *OLIG2* promoter in cortical progenitors in the human prenatal brain. Thus, we have identified a conserved molecular mechanism that promotes *Olig2/OLIG2* expression and a cortical neural stem cell lineage switch in the mouse and human.

## Results

### Shh Signaling Promotes the Generation and Proliferation of Ascl1^+^Egfr^+^ and Egfr^+^Olig2^+^ Intermediate Progenitors (IMPs) in the Cortical VZ/SVZ.

**Fig. 1.**
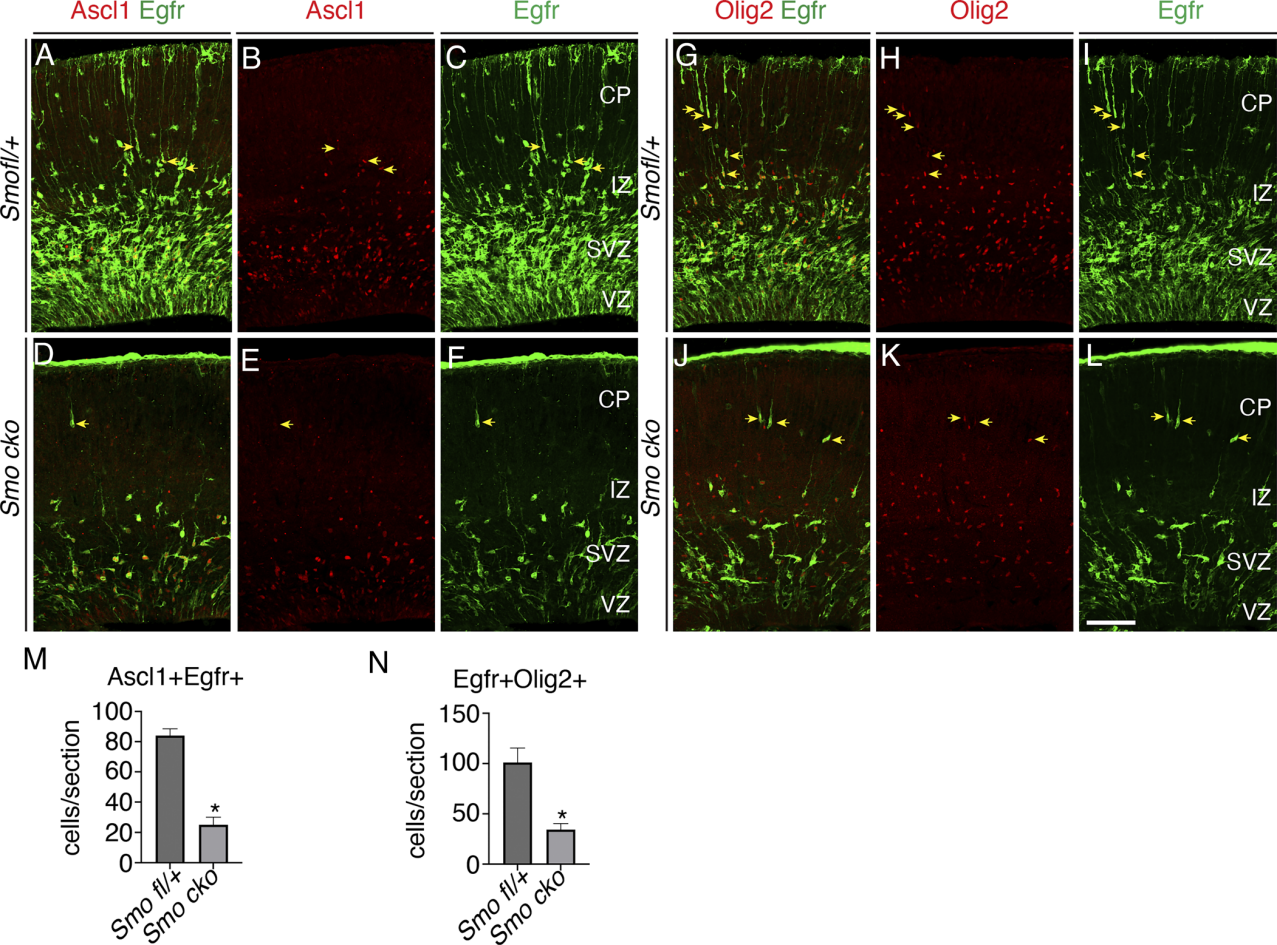
Fewer Ascl1^+^Egfr^+^ and Egfr^+^Olig2^+^ IMPs were present in the cortical VZ/SVZ of E17.5 *Smo cko* mice. (*A*–*L*) Immunostaining for Ascl1, Egfr, and Olig2 in *Smo^fl/+^* (*A*–*C* and *G*–*I*) and *Smo cko* cortices (*D*–*F* and *J*–*L*). Images were taken at the rostral-middle position along the rostral-caudal axis. (*M* and *N*) Quantification of the Ascl1^+^Egfr^+^ and Egfr^+^Olig2^+^ cells. Numbers represent means + SEM (n = 3 mice per genotype). *P < 0.05; unpaired Student’s *t* test. (Scale bars: 100 μm in *L*, applies to *A*–*L*.)

As cortical RGCs undergo the lineage switch to generate OB interneurons and cortical glia, they generate IMPs for these lineages that can be identified by coexpression of Ascl1, Egfr, and Olig2 (12, 13). We and others reported that Shh signaling is essential for cortical RGCs to produce oligodendrocytes and OB interneurons (4, 22). To determine how Shh signaling regulates this lineage switch, we deleted the Shh receptor and signaling transducer, *Smoothened* (*Smo*), in late cortical RGCs and examined the expression of Ascl1, Egfr, and Olig2 in the cortical SVZ of control (*Smo^fl/+^*) and *hGFAP-Cre; Smo^fl/fl^* (*Smo cko*) mice (23). Since both Ascl1 and Olig2 antibodies were made in rabbits, we examined the Ascl1^+^Egfr^+^ and Egfr^+^Olig2^+^ IMPs. In the control brains, the Ascl1^+^Egfr^+^ and Egfr^+^Olig2^+^ IMPs were initially observed at E16.5, and their numbers continued to increase until birth (Fig. 1 *A*–*C* and *G*–*I*). In the *Smo cko* brains however, expression of Ascl1 was reduced, and Egfr and Olig2 expressions were absent in the cortical VZ/SVZ at E16.5. Ascl1^+^Egfr^+^ and Egfr^+^Olig2^+^ cortical IMPs were observed from E17.5, but, compared to the control brains, the numbers of Ascl1^+^Egfr^+^ and Egfr^+^Olig2^+^ cells were significantly reduced (Fig. 1 *D*–*F* and *J*–*N*). Thus, blocking Shh signaling leads to delayed appearance and reduced numbers of Ascl1^+^Egfr^+^ and Egfr^+^Olig2^+^ IMPs, which give rise to cortical glia and some OB interneurons.

To examine whether Shh signaling regulates the proliferation of these IMPs, we injected EdU at E17.5 to label S phase cells and collected the brains 2 h later (*SI Appendix*, Fig. S1). We found that, compared to littermate *Smo^fl/+^* control mice, the numbers of EdU^+^, EdU^+^Olig2^+^, EdU^+^Ascl1^+^, EdU^+^Egfr^+^, EdU^+^Ascl1^+^Egfr^+^, and EdU^+^Olig2^+^Id1^+^ cells in the VZ/SVZ of *Smo cko* mice were significantly reduced (*SI Appendix*, Fig. S1 *A*–*Q*, *S*–*W*, and *Y*). The numbers of Ki67^+^ cells were also reduced (*SI Appendix*, Fig. S1 *R*, *X*, and *Y*). Quantification of the percentages of Ascl1^+^, Egfr^+^, and Olig2^+^ cells that were labeled by EdU indicated that lower percentages of Ascl1^+^ and Olig2^+^ progenitor cells were in S phase in the *Smo cko* mice (*SI Appendix*, Fig. S1*Y*). This indicates that in addition to promoting cortical RGCs to generate the IMPs for the macroglial and OB interneuron lineages, Shh signaling promotes the proliferation of these IMPs.

### Olig2 Is Essential for Cortical RGCs to Generate Oligodendrocytes, Astrocytes, and Olfactory Bulb Interneurons.

**Fig. 2.**
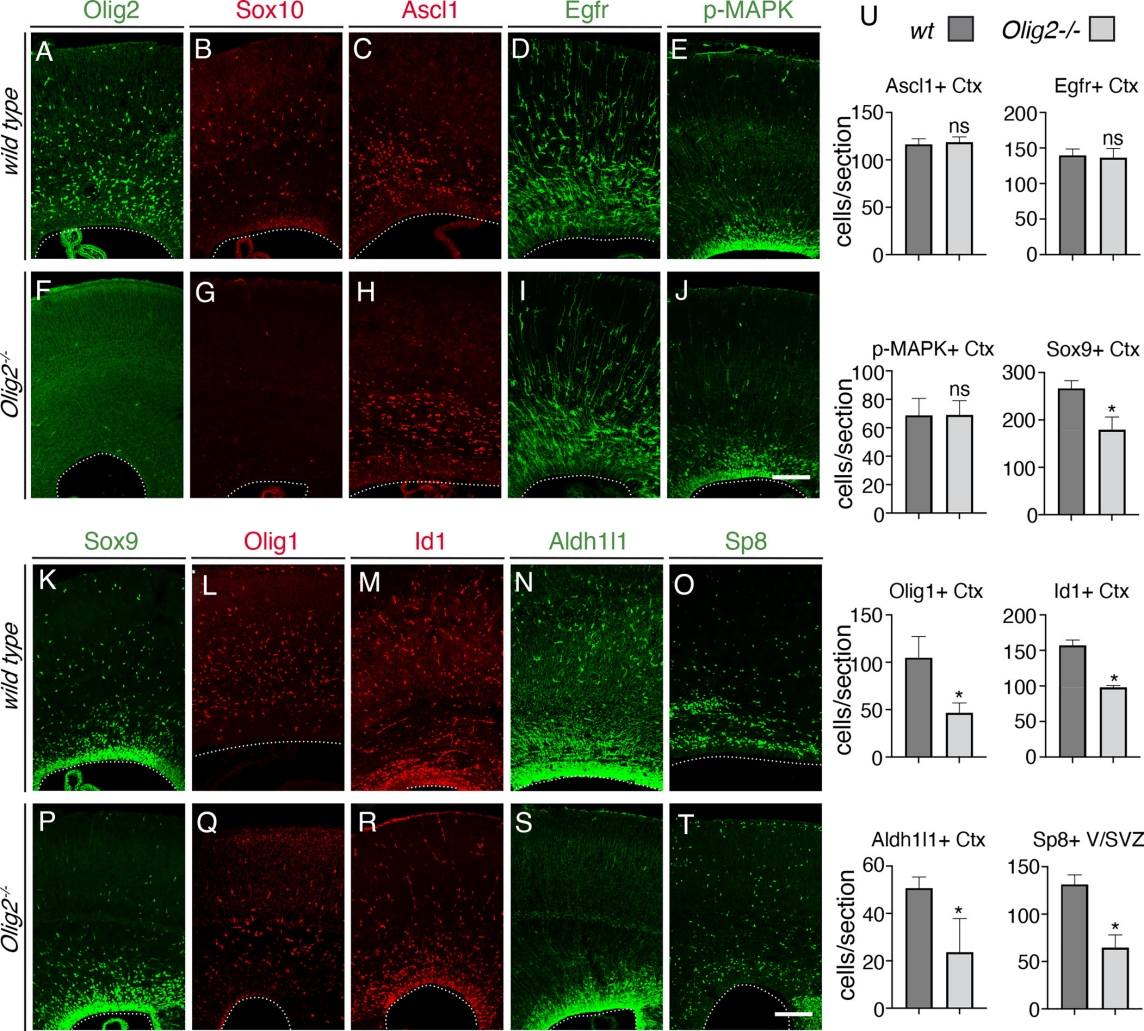
*Olig2* deletion leads to fewer cells of oligodendrocyte, astrocyte, and olfactory bulb interneuron lineages at E18.5. (*A*–*T*), Immunostaining for Olig2 (*A* and *F*), Sox10 (*B* and *G*), Ascl1 (*C* and *H*), Egfr (*D* and *I*), phosphorylated MAPK (*E* and *J*), Sox9 (*K* and *P*), Olig1 (*L* and *Q*), Id1 (*M* and *R*), Aldh1l1 (*N* and *S*), and Sp8 (*O* and *T*) in wild-type (*A*–*E* and *K*–*O*) and *Olig2^−/−^* (*F*–*J* and *P*–*T*) cortices. Images were taken at the rostral-middle position along the rostral-caudal axis. (*U*) Quantification of Ascl1^+^, Egfr^+^, phosphorylated MAPK^+^, Sox9^+^, Olig1^+^, Id1^+^, Aldh1l1^+^, and Sp8^+^ cells. Numbers represent means + SEM (n = 3 mice per genotype). *P < 0.05; unpaired Student’s *t* test. (Scale bars: 100 μm in *J* and *T*, applies to *A*−*J* and *K*−*T*, respectively.

Lineage analysis of the Olig2^+^ cortical progenitors showed that they give rise to oligodendrocytes, all cortical astrocytes, and some OB interneurons (12). We examined the function of Olig2 in the lineage switch of cortical RGCs by analyzing the *Olig2^−/−^* mice (24) at E18.5 (Fig. 2), immediately before their neonatal death. No Sox10^+^ oligodendrocyte precursors (OPCs) and oligodendrocytes were observed in the cortices of *Olig2^−/−^* mice (Fig. 2 *B* and *G*), consistent with its essential role in oligodendrocyte development. Compared to the wild-type cortices, we did not observe significant changes in the numbers of Ascl1^+^ and Egfr^+^ cells in the SVZ (Fig. 2 *C*, *D*, *H*, *I*, and *U*), indicating that the generation of Ascl1^+^ and Egfr^+^ IMPs do not depend on Olig2.

The mitogen-activated protein kinase (MAPK) pathway is essential for gliogenesis in the developing cortex (25). We examined the numbers of activated/phosphorylated MAPK^+^ cells in the SVZ and found that they were not significantly affected in the *Olig2^−/−^* mice at E18.5 (Fig. 2 *E*, *J*, and *U*), consistent with *Olig2* expression being downstream of MAPK pathway (25). The numbers of the Olig1^+^ and Sox9^+^ glial progenitors in the cortical plate were significantly reduced in the absence of *Olig2* (Fig. 2 *K*, *L*, *P*, *Q*, and *U*). Id1 and Aldh1l1 are expressed in RGCs in the VZ, IMPs in the SVZ, as well as astrocyte precursors and astrocytes in the cortical plate. Both Id1^+^ and Aldh1l1^+^ cells were significantly reduced in the SVZ and the cortical plate of *Olig2^−/−^* mice (Fig. 2 *M*, *N*, *R*, *S*, and *U*). While the numbers of Sp8^+^ cortical interneurons were not affected, the numbers of Sp8^+^ olfactory bulb interneuron neuroblasts in the SVZ were significantly reduced in the *Olig2^−/−^* mice (Fig. 2 *O*, *R*, and *U*). Thus, in addition to oligodendrocytes, Olig2 is required for cortical RGCs to generate proper numbers of astrocyte progenitors and olfactory bulb interneurons.

### Gli3 Inhibits *Olig2* Expression in the Cortical Progenitor Cells.

We recently showed that Shh signaling promotes the lineage switch of cortical RGCs by causing the degradation of transcription repressor Gli3 (4). To determine the molecular mechanism underlying *Olig2* expression in cortical progenitor cells, we examined Gli3 and Olig2 protein expression in the cortical VZ/SVZ at P0 using immunohistochemistry and western blot analysis (*SI Appendix*, Fig. S2). Cell density did not change in *Smo cko* mice (*SI Appendix*, Fig. S2 *G*, *K*, *O*, and *S*). In the *Smo cko* mice, more cells expressed Gli3 (*SI Appendix*, Fig. S2 *E*, *I*, *M*, and *Q*), and the number of Olig2^+^ progenitor cells in the cortical VZ/SVZ was reduced (*SI Appendix*, Fig. S2 *F*, *J*, *N*, and *R*). Compared to the wild-type cells, Gli3 protein in the *Smo cko* cortical progenitors shows a distribution that is significantly skewed toward higher expression (*SI Appendix*, Fig. S2*A*), and the Olig2 protein is significantly skewed toward lower expression (*SI Appendix*, Fig. S2*B*). Quantification of fluorescence levels shows a negative correlation between Gli3 and Olig2 expression in both wild-type and *Smo cko* VZ/SVZ cortical progenitor cells (*SI Appendix*, Fig. S2*C*), suggesting that Gli3 inhibits *Olig2* expression. Western blot showed that compared to the wild-type cortices, the ratio of Gli3 repressor (Gli3R) to the activator (Gli3A) in the P0 *Smo cko* cortices was significantly increased (*SI Appendix*, Fig. S2 *T* and *U*), further supporting Shh signaling promotes *Olig2* expression by decreasing Gli3R.

### Gli3 and Pax6 Regulate OB Interneuron Production from Cortical Progenitors Combinatorially.

**Fig. 3.**
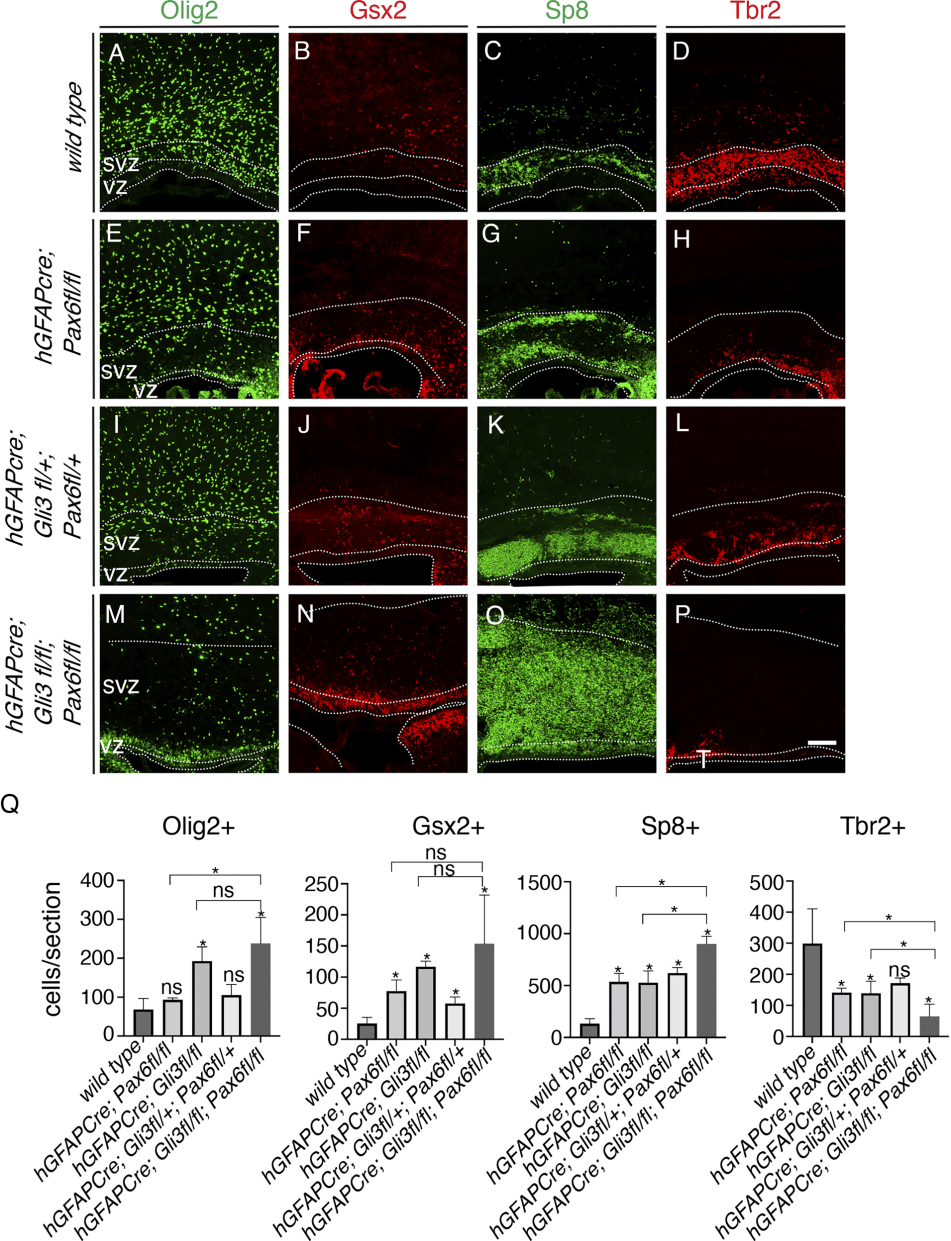
Pax6 and Gli3 repress olfactory bulb interneuron lineage. (*A*–*P*), Immunostaining of Olig2 (*A*, *E*, *I*, and *M*), Gsx2 (*B*, *F*, *J*, and *N*), Sp8 (*C*, *G*, *K*, and *O*), and Tbr2 (*D*, *H*, *L*, and *P*) in the cortices of P0 wild-type (*A*–*D*), *hGFAPcre; Pax6^fl/fl^* (*E*–*H*)*, hGFAPcre; Gli3^fl/+^; Pax6^fl/+^* (*I*–*L*), and *hGFAPcre; Gli3^fl/fl^; Pax6^fl/fl^* (*M*–*P*) mice. Images were taken at the rostral-middle position along the rostral-caudal axis. (*Q*) Quantification of Olig2^+^, Gsx2^+^, Sp8^+^, and Tbr2^+^ cells in 300-μm wide VZ/SVZ regions. Dotted lines demarcate the VZ and SVZ. Numbers represent means + SEM (n = 3 mice per genotype). ns, not significant; *P < 0.05; unpaired Student’s *t* test. (Scale bar: 100 μm in *P*, applies to *A*–*P*.)

Pax6 is highly expressed in cortical RGCs. Its expression in glial progenitors of the neonatal SVZ represses Olig2 expression and induces a neurogenic fate (26). To determine whether Pax6 regulates gliogenesis and OB interneuron production and to circumvent the early requirement of Pax6 in regulating the dorsal–ventral patterning of the telencephalon (27), we examined the brains of E16.5 and P0 *hGFAP-Cre*; *Pax6^fl/fl^* and *Emx1-Cre*; *Pax6^fl/fl^* mice (*SI Appendix*, Fig. S3). Immunostaining with cortical projection neuron markers Ctip2 and Satb2 indicated that dorsal–ventral patterning of the telencephalon occurred normally in these mice (*SI Appendix*, Fig. S3 *P*–*U*). Compared to the wild-type mice, significantly more Olig2^+^, Gsx2^+^, and Sp8^+^ cells, and fewer Tbr2^+^ excitatory neuron progenitors were present in the VZ/SVZ of E16.5 *hGFAP-Cre*; *Pax6^fl/fl^* and *Emx1-Cre*; *Pax6^fl/fl^* cortices (*SI Appendix*, Fig. S3 *A*–*O* and *V*), indicating that similar to Gli3 (4), Pax6 promotes excitatory neuron lineage, and inhibits cortical RGCs from generating glia and OB interneurons. At P0, while Gsx2^+^ cortical progenitors and Sp8^+^ OB interneuron neuroblasts remained increased in the *hGFAP-Cre*; *Pax6^fl/fl^* mice (Fig. 3 *B*, *C*, *F*, *G*, and *Q*), the number of Olig2^+^ cells in the cortical VZ/SVZ was no longer significantly increased (Fig. 3 *A*, *E*, and *Q*), likely due to the inhibition by high-level Gsx2 expression.

The similar lineage defects in the VZ/SVZ of *hGFAP-Cre*; *Pax6^fl/fl^* (*SI Appendix*, Fig. S3) and the *hGFAP-Cre*; *Gli3^fl/fl^* mice (4) indicate that both Pax6 and Gli3 inhibit cortical RGCs to generate olfactory bulb interneurons and cortical glia. To investigate whether they act synergistically, we analyzed brains from P0 *hGFAP-Cre*; *Gli3^fl/+^*; *Pax6^fl/+^* and *hGFAP-Cre*; *Gli3^fl/fl^*; *Pax6^fl/fl^* mice (Fig. 3). Neither *hGFAP-Cre*; *Gli3^fl/+^* nor *hGFAP-Cre*; *Pax6^fl/+^* mice showed changes in the Olig2^+^, Gsx2^+^, or Sp8^+^ cells in the cortical VZ/SVZ. Compared to the wild-type mice (Fig. 3 *A*–*C*), the numbers of Olig2^+^ cortical progenitor cells were not significantly affected, but the numbers of Gsx2^+^ cortical progenitor cells and the Sp8^+^ OB interneuron neuroblasts were significantly increased in the cortical VZ/SVZ of P0 *hGFAP-Cre*; *Gli3^fl/+^*; *Pax6^fl/+^* mice (Fig. 3 *I*–*K* and *Q*). In the P0 *hGFAP-Cre*; *Gli3^fl/fl^*; *Pax6^fl/fl^* brains, the numbers of Gsx2^+^ IMPs in the cortical VZ/SVZ were not significantly different from those in the *hGFAP-Cre*; *Pax6^fl/fl^* and the *hGFAP-Cre*; *Gli3^fl/fl^* mice, but the number of the Sp8^+^ OB interneuron neuroblasts drastically increased (Fig. 3 *M*–*O* and *Q*). These results indicate that *Gli3* and *Pax6* function in parallel to inhibit cortical RGCs from generating OB interneuron lineage. Consistent with this, protein coimmunoprecipitation with either a Gli3 antibody or a Pax6 antibody did not pull down the other protein.

### Identification of Genome-wide Gli3 Binding Sites in the Cortical Cells.

To determine whether Gli3 inhibits *Olig2* expression directly, we performed chromatin-immunoprecipitation (ChIP) and Cleavage Under Targets & Release Using Nuclease (CUT&RUN) using a Gli3 antibody and dissected E15 cortical tissues, followed by high-throughput DNA sequencing (28). The results were highly consistent both within and between the ChIP-seq and CUT&RUN experiments (n = 3 for each experiment). Both ChIP and CUT&RUN showed the specific binding of Gli3 to promoters and enhancers of Shh target genes such as *Gli1* and *Ptch1* (*SI Appendix*, Fig. S4). We identified 4,414 Gli3 binding sites (GBS) in the ChIP-seq experiments using irreproducible discovery rate (IDR) analysis (29). The GBS are located in both promoter and nonpromoter regions (*SI Appendix*, Fig. S5*A*). We identified the motifs enriched in the GBS at the promoter and nonpromoter regions using the MEME-ChIP online tool, and the most significantly enriched motifs were the known consensus Gli binding sequences (*SI Appendix*, Fig. S5*B*).

To determine whether the GBS were potential promoters or enhancers, we performed CUT&RUN for H3K27me3, H3K27ac, and H3K4me3 using E15 and E16 cortices. We intersected the GBS at promoter [from transcription start site (TSS) and up to 2 kb upstream] regions and nonpromoter [more than 2 kb away from the TSS] regions, with the binding sites for H3K27me3, H3K27ac, and H3K4me3 revealed in our CUT&RUN data. We found that the GBS at both the promoter and nonpromoter regions were enriched with these histone modifications (*SI Appendix*, Fig. S5 *C* and *D*), indicating that the identified GBS are likely active promoters and enhancers.

### Gli3 and Pax6 Bind to Conserved Sequences in the *Olig1/2* Loci.

**Fig. 4.**
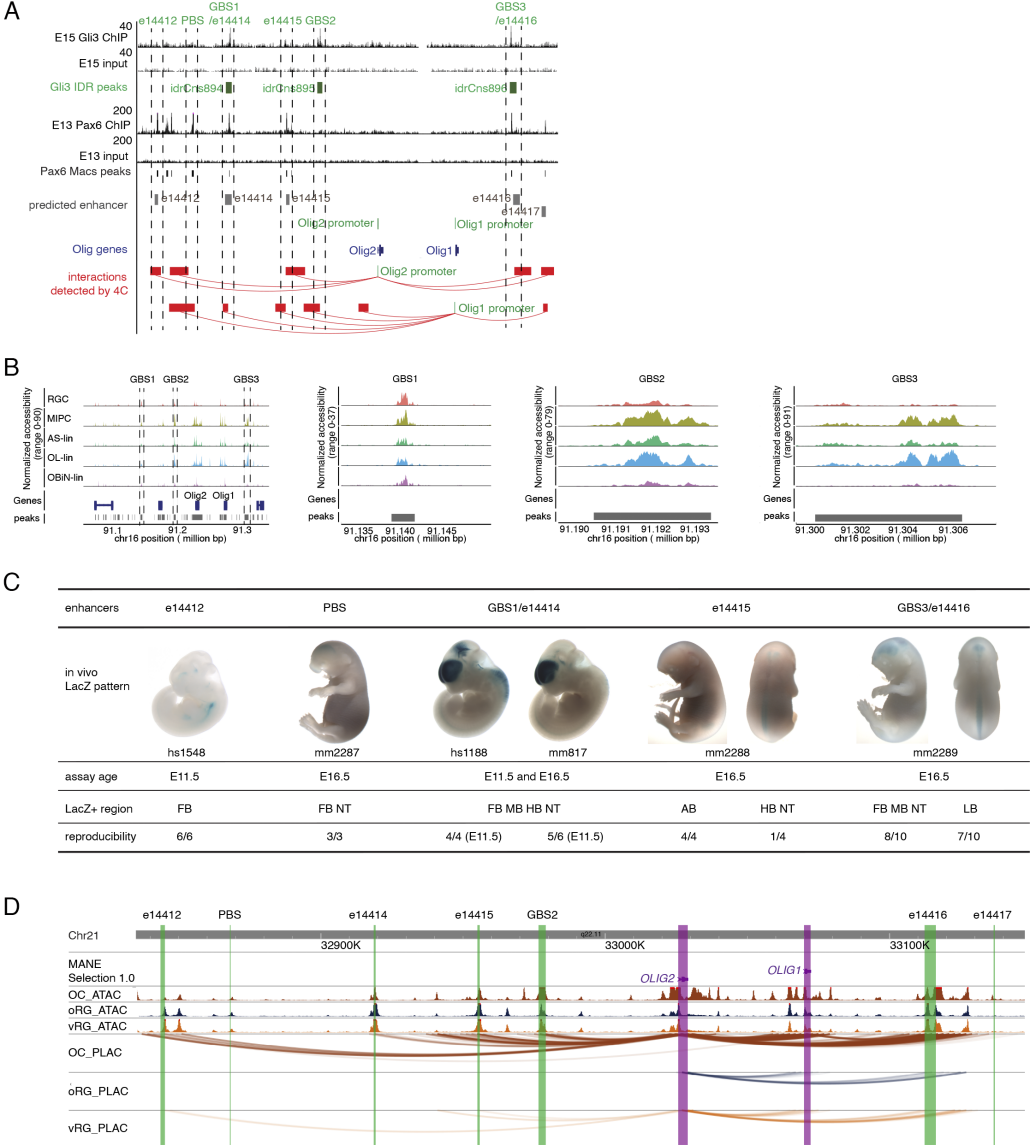
Gli3 and Pax6 binding sites in the *Olig1/2* loci have enhancer activity and their interactions with the *Olig1/2* promoters are conserved between the mouse and human. (*A*) ChIP-seq analysis showed Gli3 binds to three sites in the *Olig1/2* loci (GBS1/e14414, GBS2, and GB3/e14416), and Pax6 binds to multiple sites including e14412, PBS, e14414, e14415, and e14416. The e14412, e14414, e14415, e14416, and e14417 are enhancers predicted by the ENCODE project, and are shown in gray. The red blocks represent the regions that interacted with *Olig2* or *Olig1* promoters in the cortical cells, as revealed in the 4C experiments. Note that GBS1 is part of e14414, and GBS3 overlaps with e14416. (*B*) Single-cell ATAC-seq revealed that the 3 GBS sequences are differentially accessible in RGCs, MIPCs, astrocyte, oligodendrocyte, and olfactory bulb interneuron lineages. (*C*) Enhancer reporter assays showed that the Gli3 and Pax6 binding sites have enhancer activity in E11.5 or E16.5 mouse embryos. Note that hs1528 (e14412) and hs1188 (e14414) are human sequences, and mm817 is the mouse sequence for e14414. All the enhancer data have been loaded in the Vista Enhancer browser: mm2287 (PBS), mm2288 (e14415), and mm2289 (e14416). Abbreviations: FB, forebrain; NT, neural tube; MB, midbrain; HB, hindbrain; AB, abdomen; LB, limb. (*D*) H3K4me3 PLAC-seq experiments revealed that the Gli3 and Pax6 binding sites interact with *OLIG1* and *OLIG2* promoters in human fetal cortical glial lineage cells. The *OLIG1* and *OLIG2* promoters are shown in pink bars, the green bars represent the homologous sequences of the Gli3 and Pax6 binding peaks in human cells, and the wavy lines represent the interactions detected in H3K4me3 PLAC-seq. ATAC peaks in OC, oRG, and vRG cells are also shown. OC, glial lineage cells; oRG, outer radial glial cells; vRG, ventricular radial glial cells.

*Olig2* and its close homolog *Olig1* are located 36 kb apart on chromosome 16 in the mouse genome. ChIP-seq revealed 3 GBS in highly conserved nonpromoter regions (Fig. 4*A* and *SI Appendix*, Fig. S6). GBS1 was within the predicted ENCODE (30–32) enhancer e14414 in brain tissue, and GBS3 overlapped with predicted enhancer e14416 (Fig. 4*A*). GBS2 and GBS3 exhibited enrichment of H3K27me3 and H3K4me3 (*SI Appendix*, Fig. S6). To further investigate whether these GBS are potential enhancers, we performed CUT&RUN for H3K27me3 and H3K4me3 using dissected cortices from E16.5 control (*RosaSmoM2*), or *hGFAP-Cre; RosaSmoM2* mice, the latter of which express a constitutively activated Smoothened protein and exhibit increased Shh signaling in cortical progenitor cells (4). We previously showed increased numbers of Olig2^+^ cortical progenitors and OB interneuron lineage cells in the VZ/SVZ of *hGFAP-Cre; RosaSmoM2* mice (4). We observed increased H3K4me3 at GBS1 and GBS3, and reduced H3K27me3 at GBS2 in the *hGFAP-Cre; RosaSmoM2* cells (*SI Appendix*, Fig. S6). In addition, we coelectroporated *pCAG-ShhN* and *pCAG-Cre* plasmids or *pCAG-Cre* plasmids alone into the cortical VZ of E13.5 *Rosa26^RCE-GFP^* reporter mice to label late cortical RGCs, and enriched for the GFP^+^ cells at E16.5 using fluorescence activate cell sorting (FACS). We performed Assay of Transposase Accessible Chromatin sequencing (ATAC-seq) on the sorted cells, and observed increased accessibility at both *Olig1* and *Olig2* genes, as well as the 3 GBS in the cells from the brain electroporated with *pCAG-ShhN* plasmids (*SI Appendix*, Fig. S6), confirming Shh signaling promotes the accessibility of *Olig1/2* genes and these GBS. To determine whether *Olig1* or *Olig2* promoters could be regulated by these GBS, we performed a 4C experiment (33), a derivative of chromatin conformation capture (3C), using the promoter sequences of *Olig1* and *Olig2* as baits to capture their interacting sequences. We found that all 3 GBS interacted with these promoters in the cortical cells (Fig. 4*A*), suggesting a direct regulatory relationship.

The *hGFAP-GFP* transgenic mouse line expresses GFP under the control of the human GFAP promoter in cortical RGCs, and the GFP proteins carry over from RGCs to their immediate progeny (34). We mined the single-cell ATAC-seq (scATAC-seq) data of 4721 *hGFAP-GFP^+^* cortical cells enriched by FACS from E18.5 *hGFAP-GFP* mice (12), and analyzed chromatin accessibility in the clusters of RGCs, MIPCs, projection neuron lineage, astrocyte lineage, oligodendrocyte lineage, and OB interneuron lineage (figure 8 in ref. 12). We found that the 3 GBS overlapped with the scATAC-seq peaks that were differentially accessible in different cell types/lineages (Fig. 4*B*), further suggesting that the GBS are likely enhancers for the *Olig1/2* genes.

To investigate how Pax6 regulates *Olig2* expression, we examined the previously published Pax6 ChIP-seq data (35) and found that Pax6 bound to several regions in the *Olig1/2* loci, including GBS1/e14414, GBS3/e14416, e14412, and e14415 (Fig. 4*A*). 4C experiments showed that the Pax6 binding sites (PBS) interacted with the *Olig1/2* promoters in the cortical cells (Fig. 4*A*). Thus, our results demonstrate that Pax6 directly represses *Olig1/2* expression in cortical progenitors.

### Gli3 Binding Sites near the *Olig1/2* Loci Show Enhancer Activity in the Developing Cerebral Cortex.

We tested whether the Gli3 and Pax6 binding sites near the *Olig1/2* loci are potential enhancers by examining the Vista Enhancer Browser (36) and generating additional reporter mice (36–38). β-galactosidase (LacZ) expression was assayed in the E11.5 or E16.5 embryos (Fig. 4*C*). The activity of the human homologous sequence (hs1548) for predicted enhancer e14412 was previously assayed by the Vista Enhancer project, and showed low but consistent (6/6) activity in the forebrain of E11.5 embryos (Fig. 4*C*). We assayed the activity of both the mouse GBS1/e14414 enhancer (mm817 in Fig. 4*C*) and the human homologous sequence (hs1188). Both the mouse and human sequences drove high and consistent expression of LacZ in the forebrain, midbrain, hindbrain, and the neural tube (Fig. 4*C*). 4C experiment showed that the Pax6 binding site between e14412 and e14414 interacted with the *Olig1/2* promoters (PBS in Fig. 4*A*). We found that the PBS was active in the forebrain and neural tube in the E16.5 mouse embryos (mm2287 in Fig. 4*C*). We observed LacZ activities in the forebrain, midbrain, neural tube, and limb for the GBS3/e14416 enhancer (mm2289) at E16.5, while e14415 (mm2288) showed activities in the hindbrain, neural tube, and the abdomen at E16.5 (Fig. 4*C*). Thus, most of the Gli3 and Pax6 binding sites at the *Olig1/2* loci showed enhancer activity in the developing brain.

### Gli3 and Pax6 Binding Sites Are Conserved in the Human Genome and Are Recruited to the *OLIG1* and *OLIG2* Promoters in the Cortical Glial Lineages.

During human prenatal brain development, cortical radial glial cells (RGCs) generate macroglia and OB interneurons via *OLIG2*-expressing IMPs (13). We characterized *cis*-regulatory chromatin interactions for RGCs and glial progenitors in fetal cortices. Briefly, we isolated cortical cells from gestation week 15 (GW15) and GW22 human cortices. These cells were dissociated, permeabilized, and stained with markers SOX2, HOPX, OLIG2, and PU.1 to allow type-specific cell isolation via fluorescence-activated cell sorting (FACS). Specifically, we targeted ventricular radial glia (vRG, SOX2^+^HOPX^low^), outer radial glia (oRG, SOX2^+^HOPX^high^), glial progenitors (IMP/OPC/oligodendrocytes, OLIG2^+^), and microglia (PU.1^+^). Following cell sorting, we performed H3K4me3 proximity ligation-assisted ChIP-seq (PLAC-seq) using sorted cells, and applied the Model-based Analysis of PLAC-seq (MAPS) pipeline to call significant H3K4me3-mediated chromatin interactions at a resolution of 2 kb (39). To determine whether the Gli3 and Pax6 binding sites identified in the cortical progenitor cells in the mouse are potential enhancers for the human *OLIG1/2* genes in cortical progenitors, we identified the human homologous sequences for these binding sites using the Basic Local Alignment Search Tool (40). We examined H3K4me3 PLAC-seq data and found that human homologous sequences for the e14412, GBS1/e14414, e14415, GBS2, GBS3/e14416, and the PBS interact with *OLIG2* or *OLIG1* promoters (Fig. 4*D*). Reporter assays showed that the human hs1188 enhancer, which is homologous to the GBS1/e11414, drives high lacZ expression in the E11.5 mouse cortex (Fig. 4*C*), suggesting that the molecular mechanism for activating *OLIG2* expression in cortical progenitor cells is conserved between the mouse and human.

### GBS1/e14414 and GBS2 Are Essential for *Olig2* Expression in Cortical Progenitors and Gliogenesis.

**Fig. 5.**
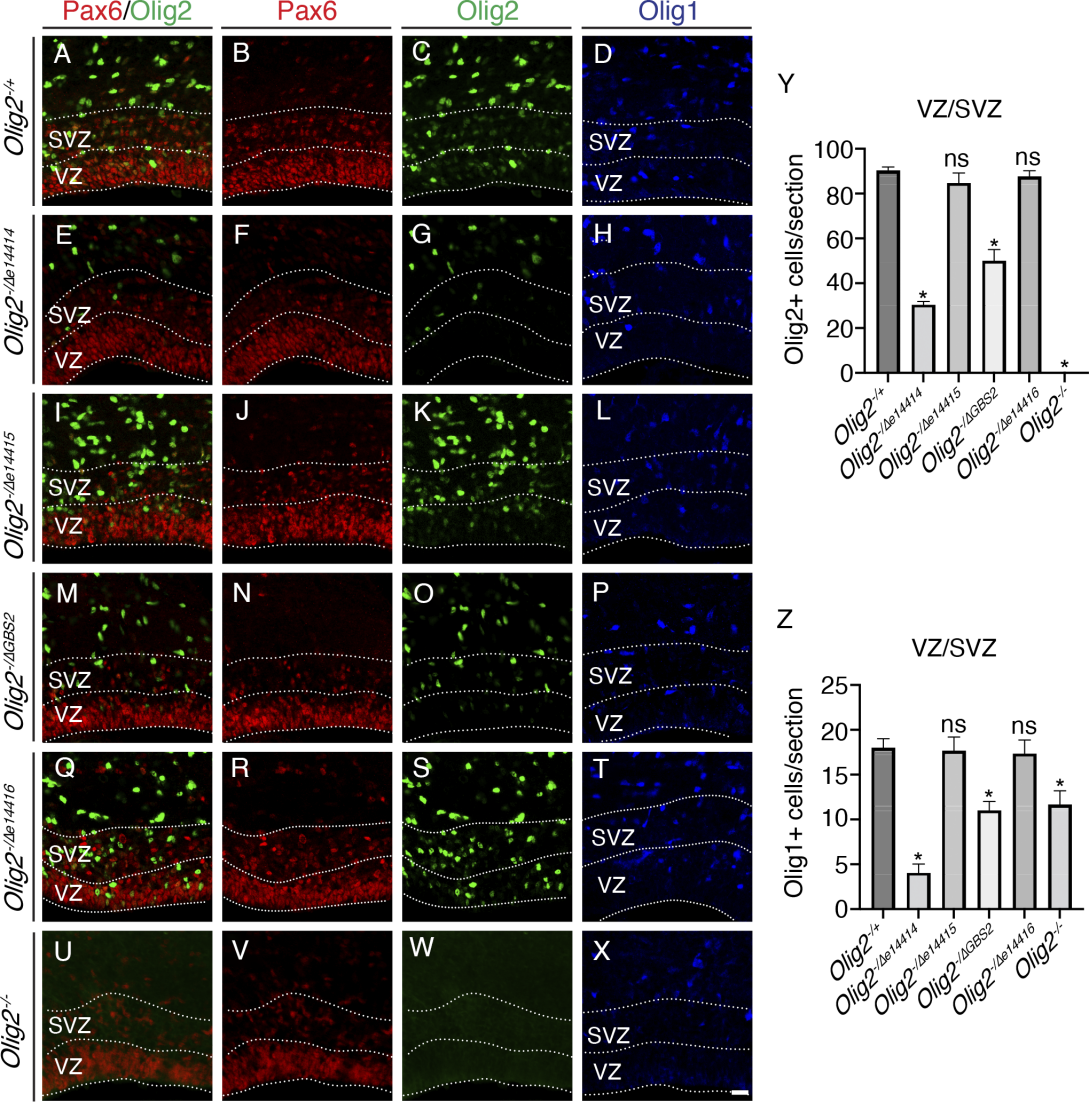
Enhancers e14414 and GBS2 regulate *Olig2* and *Olig1* expression in cortical VZ/SVZ. P0 images were shown. (*A*–*X*) Immunostaining for Pax6, Olig2, Olig1 in *Olig2^−/+^* (*A*–*D*), *Olig2^−/Δe14414^* (*E*–*H*), *Olig2^−/Δe14415^* (*I*–*L*), *Olig2^−/ΔGBS2^* (*M*–*P*), *Olig2^−/Δe14416^* (*Q*–*T*), and *Olig2^−/−^* (*U*–*X*) cortices. Pax6 expression delineates the VZ and SVZ. Images were taken at the rostral-middle position along the rostral-caudal axis. (*Y* and *Z*) Quantification Olig2^+^ and Olig1^+^ cells in 350-μm wide cortical VZ/SVZ regions. Numbers represent means + SEM (n = 3 mice per genotype). *P < 0.05; unpaired Student’s *t* test. (Scale bar: 20 μm in *X*, applies to *A*–*X*.)

To determine whether the Gli3 and Pax6 binding sites are necessary for *Olig2* expression in cortical progenitor cells, we generated mice carrying deletion alleles for GBS1/e14414, e14415, GBS2, and GBS3/e14416 using a CRISPR-Cas9 strategy (*SI Appendix*, Fig. S7). We analyzed P0 brains from homozygous enhancer deletion mutant mice (*SI Appendix*, Fig. S7). In the *Olig2^Δe14414/Δe14414^*, *Olig2^ΔGBS2/ΔGBS2^*, and *Olig2^Δe14415/Δe14415^* mice, Olig2^+^ and Olig1^+^ cell numbers in the cortical VZ/SVZ were reduced, while their numbers were not significantly affected in the *Olig2^Δe14416/Δe14416^* mice (*SI Appendix*, Fig. S7). To circumvent effects of potential secondary mutations carried by the enhancer deletion alleles, we bred mice carrying enhancer deletion alleles to mice carrying an *Olig2* null allele (*Olig2^−^*) (24) and analyzed brains from trans-heterozygous mice carrying one copy of an enhancer deletion allele and one copy of the *Olig2^−^* allele. We compared the trans-heterozygotes to the *Olig2^+/−^* and *Olig2^−/−^* mice (Fig. 5). We found that Olig2^+^ cells were present in the VZ/SVZ and cortical plate in the *Olig2^+/−^*, *Olig2^−/Δe14414^*, *Olig2^−/Δe14415^*, *Olig2^−/ΔGBS2^*, and the *Olig2^−/Δe14416^* mice (Fig. 5 *C*, *G*, *K*, *O*, and *S*), and absent in the *Olig2^−/−^* mice (Fig. 5*W*). Compared to the *Olig2^+/−^* mice, the numbers of Olig2^+^ cells in the VZ/SVZ were significantly reduced in the *Olig2^−/Δe14414^* and *Olig2^−/ΔGBS2^*mice, but not in the *Olig2^−/Δe14415^*and *Olig2^−/Δe14416^* mice (Fig. 5*Y*), indicating that e14414 and GBS2, but not e14415 or e14416, are essential for efficient Olig2 expression in the cortical progenitor cells. Similarly, the numbers of Olig1^+^ cells in the VZ/SVZ were reduced in both *Olig2^−/Δe14414^* and *Olig2^−/ΔGBS2^* mice (Fig. 5 *D*, *H*, *P*, and *Z*).

Olig2 is expressed in the lateral and medial VZ/SVZ, as well as the ventral VZ/SVZ. We observed reduced numbers of Olig2^+^ cells in the lateral, but not medial or ventral VZ/SVZ in the *Olig2^−/Δe14414^* and *Olig2^Δe14414/Δe14414^* mice (*SI Appendix*, Fig. S8). Olig2 expression in the lateral, medial, or ventral VZ/SVZ was not affected in the *Olig2^Δe14415/Δe14415^*, *Olig2^ΔGBS2/ΔGBS2^*, or the *Olig2^Δe14416/Δe14416^* mice (*SI Appendix*, Fig. S8). Thus, e14414 regulates *Olig2* expression in the lateral VZ/SVZ.

**Fig. 6.**
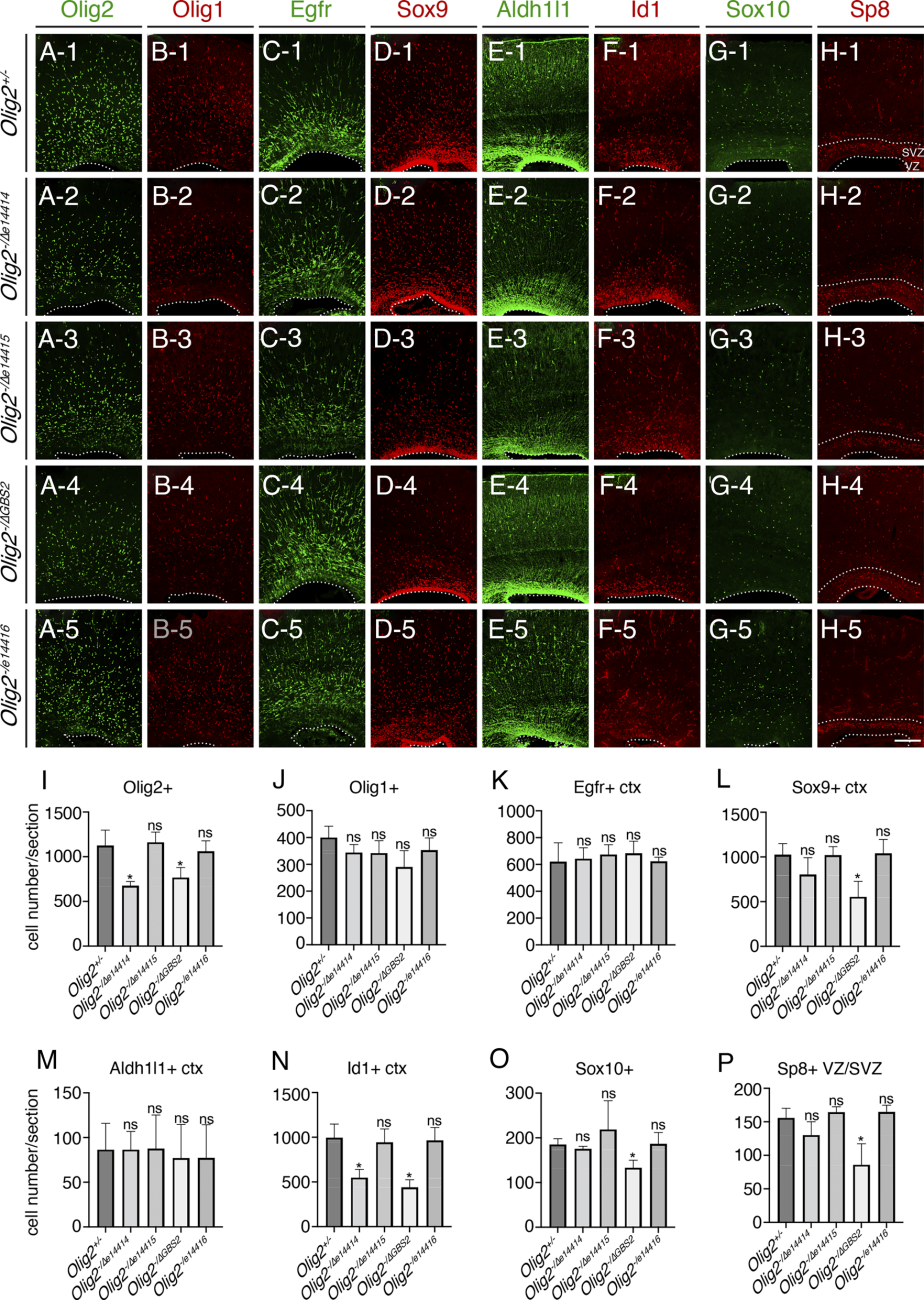
Deletion of e14414 or GBS2 leads to defective lineage switch in the cortex. P0 images were shown. (*A*–*H*) Immunostaining for Olig2 (*A*-1 to *A*-5), Olig1 (*B*-1 to *B*-5), Egfr (*C*-1 to *C*-5), Sox9 (*D*-1 to *D*-5), Aldh1l1 (*E*-1 to *E*-5), Id1 (*F*-1 to *F*-5), Sox10 (*G*-1 to *G*-5), and Sp8 (*H*-1 to *H*-5) in the cortices of *Olig2^+/−^* (*A*-1 to *H*-1), *Olig2^−/Δe14414^* (*A*-2 to *H*-2), *Olig2^−/Δe14415^* (*A*-3 to *H*-3), *Olig2^−/ΔGBS2^* (*A*-4 to *H*-4), and *Olig2^−/Δe14416^* (*A*-5 to *H*-5) mice. Images were taken at the rostral-middle position along the rostral-caudal axis. (*I*–*P*) Quantification Olig2^+^, Olig1^+^, Egfr^+^, Sox9^+^, Aldh1l1^+^, Id1^+^, Sox10^+^, and Sp8^+^ cells. Numbers represent means + SEM (n = 3 mice per genotype). *P < 0.05; unpaired Student’s *t* test. (Scale bar: 100 μm in *H*-5, applies to *A*-1 to *H*-5.)

We determined whether deletion of any of these enhancers affected gliogenesis or OB interneuron production in the cortex using markers for glial progenitors, OB interneuron progenitors, and immature OB interneurons (Fig. 6). At P0, the Olig2^+^, Olig1^+^, Egfr^+^, and Sox9^+^ cells in the cortical plates are glial progenitors that can generate both astrocytes and oligodendrocytes. Compared to the *Olig2^+/−^* mice, the numbers of Olig2^+^ cells in the cortical plate were significantly reduced in the *Olig2^−/Δe14414^* and *Olig2^−/ΔGBS2^* mice, but not in the *Olig2^−/Δe14415^* or *Olig2^−/Δe14416^* mice (Fig. 6*I*). The numbers of Sox9^+^ cells were decreased in the cortical plate of the *Olig2^−/ΔGBS2^* mice (Fig. 6*L*), while the numbers of Olig1^+^ and Egfr^+^ cells in the cortical plate were not significantly affected in the enhancer trans-heterozygous deletion mice (Fig. 6 *J* and *K*). Aldh1l1 and Id1 are mostly expressed in astrocyte progenitors in the cortical plate at P0. Aldh1l1^+^ cells in the cortical plate in the enhancer trans-heterozygous deletion mice were not significantly different from those in the *Olig2^+/−^* mice (Fig. 6*M*), while the Id1^+^ cells in the cortical plate showed a significant reduction in the *Olig2^−/Δe14414^* and *Olig2^−/ΔGBS2^* mice (Fig. 6*N*). There were fewer Sox10^+^ oligodendrocytes precursor cells in the cortical plate in the *Olig2^−/ΔGBS2^* mice, but not in other enhancer trans-heterozygous deletion mice (Fig. 6*O*). Sp8 expression marks the OB interneuron progenitors and immature OB interneurons in the cortical VZ/SVZ. Compared to the *Olig2^+/−^* mice, the numbers of Sp8^+^ OB interneuron precursors were significantly reduced in the *Olig2^−/ΔGBS2^* mice (Fig. 6*P*). Together, these results indicate that enhancers e14414 and GBS2 are both required for efficient expression of *Olig2* in the cortical progenitors, while deletion of e14414 reduced the Id1^+^ glial progenitors, and deletion of GBS2 reduced the progenitors for both types of cortical macroglia and olfactory bulb interneuron precursors. Thus, GBS2 is essential for cortical gliogenesis and OB interneuron production.

## Discussion

At the end of cortical excitatory neuron production, cortical RGCs switch lineage to generate astrocytes, oligodendrocytes, and OB interneurons (3–6, 10, 12). Some late cortical RGCs migrate away from the VZ into the cortical plate; they become translocating RGCs that produce astrocytes. Other late RGCs divide at VZ and generate transient Ascl1^+^Egfr^+^Olig2^+^ MIPCs; these MIPCs divide and generate the progenitors for cortical astrocytes, oligodendrocytes, and inhibitory OB interneurons (12, 13). Immunohistochemical and scRNA-seq data analyses revealed the presence of Ascl1^+^Egfr^+^Olig2^+^ MIPCs in the cortical VZ/SVZ of fetal human brains, which give rise to intermediate progenitors for both cortical macroglia and OB interneurons (13). This indicates that the generation of Ascl1^+^Egfr^+^Olig2^+^ cortical MIPCs and activating expression of Egfr and Olig2 in cortical progenitors are conserved during mammalian brain development.

Recent elegant studies revealed the important functions of Egfr in regulating cortical gliogenesis (5, 6). Using mosaic analysis with double markers (MADM) to sparsely delete Egfr in cortical progenitors, Zhang et al. showed Egfr to be required in rostrodorsal, but not ventrocaudal glial lineages (5, 6). Lineage analysis of Olig2^+^ cortical progenitors indicated that they give rise to OB interneurons, cortical oligodendrocytes, and almost all the cortical astrocytes (12). Expression of *Olig2* in the cortical progenitors is essential for cortical RGCs to generate astrocytes and oligodendrocytes (18, 21). During mouse embryonic and human fetal brain development, both *Egfr/EGFR* and *Olig2/OLIG2* are initially expressed in the VZ/SVZ of the ventral forebrain and start to be expressed in the cortical VZ/SVZ as cortical RGCs start to generate glial and OB interneuron lineages (12, 13). How *Egfr*/EGFR** and *Olig2*/OLIG2** become transcriptionally activated in cortical progenitors has remained unexplored.

In this study, we showed that expression of both *Egfr* and *Olig2* in cortical progenitors is under the control of Shh signaling. We focused on how the transcription of *Olig2* is regulated in cortical progenitors by identifying its cortical enhancers. We showed that Gli3 and Pax6, two transcription factors highly expressed in cortical RGCs, inhibit *Olig2* expression. By performing ChIP and CUT&RUN experiments and examining previously published Pax6 ChIP-seq data (35), we showed that both Gli3 and Pax6 bind to multiple sites in the *Olig1/2* loci (Fig. 4*A* and *SI Appendix*, Fig. S6). Results from CUT&RUN experiments using antibodies for H3K4me3, H3K27me3, and H3K27ac as well as bulk ATAC-seq and single-cell ATAC-seq experiments suggested that the Gli3 and Pax6 binding sites in the *Olig1/2* loci were potential enhancers (Fig. 4 *A* and *B* and *SI Appendix*, Fig. S6). 4C experiments showed that Gli3 and Pax6 binding sites interacted with *Olig1/2* promoters in the developing cortex (Fig. 4*A*). Transgenic reporter mice showed that e14412, PBS, e14414/GBS1, e14415, and e14416/GBS3 had enhancer activity (Fig. 4*B*), and some of them were active in the developing forebrain (Fig. 4*B*). By generating enhancer knockout mice, we showed that both e14414/GBS1 and GBS2 are essential for efficient *Olig2* expression in the cortical progenitors, and are required for proper generation of glial progenitors in the developing cortex (Figs. 5 and 6). Thus, we have identified critical enhancer sequences for *Olig2* transcriptional regulation.

Cortical RGC lineage switch ensures proper numbers of neuronal and glial cell types are generated, and it is a conserved, but incompletely understood, feature in mouse and human cortical development (12, 13). Similar to the mouse brain, *OLIG2* expression begins in the human cortical SVZ when cortical progenitors start to generate glial lineages in the fetal brain (13). We found that the sequences of Gli3 and Pax6 binding sites are conserved in the human genome, and they interact with the *OLIG2* and *OLIG1* promoters in the cortical progenitor cells and glial lineages in the fetal brain (Fig. 4*D*). Similar to the mouse e14414/mm817 sequence, the homologous human sequence hs1188 drives strong LacZ expression in the mouse embryonic cortex (Fig. 4*C*). These results reveal conserved regulatory logic for *OLIG2* expression during human brain development.

Although we have identified critical enhancers that regulate *Olig2* expression in cortical progenitors, the molecular mechanism for how *Egfr* is transcriptionally activated by Shh and other signaling pathways remains to be determined. Interestingly, while Shh signaling is required across the rostro-caudal and medial-lateral axes of the cortical VZ/SVZ to activate *Olig2* and *Egfr* expression, *Egfr* regulates cortical gliogenesis in a regional-dependent manner (5, 6). Given that the MAPK pathway is strictly required for cortical gliogenesis (25), it is likely that signal transduction pathways other than the Egfr signaling activate the MAPK pathway in the ventrocaudal cortical regions.

Oligodendrocytes in the cerebral cortex originate from multiple sources (41). Although Ascl1^+^Egfr^+^Olig2^+^ MIPCs and Olig2^+^ Sox10^+^ OPCs are reduced in the cortices of *Smo cko* around birth, the numbers of OPCs and oligodendrocytes partially recover over time during later developmental stages (42). Both the expansion of ventrally derived OPCs and the proliferation of remaining cortical-derived OPCs contribute to this partial recovery (42). Future study should address the mechanisms by which the cortical RGCs generate OPCs and oligodendrocytes in the absence of Shh signaling.

## Materials and Methods

Experiments were performed according to protocols approved by the Institutional Animal Care and Use Committee at University of California at Santa Cruz, University of South Dakota Sanford School of Medicine, and Lawrence Berkeley National Laboratory. Deidentified fetal tissue samples were collected with prior informed consent in strict observance of legal and institutional ethical regulations. All protocols were approved by the Human Gamete, Embryo, and Stem Cell Research Committee and Institutional Review Board at the University of California, San Francisco.

Generation of *Olig2^Δe14415/+^*, *Olig2^ΔGBS2/+^*, and *Olig2^Δe14416/+^* mice: These mice were generated using the iGONAD method (43), using two guide RNAs and a HDR donor oligo as repair template for each enhancer. Detailed information can be found in *SI Appendix*.

Generation of the enhancer reporter mice: These mice were generated and analyzed as described previously (36–38). Detailed information can be found in *SI Appendix*.

Immunohistochemistry, ChIP-seq, CUT&RUN, ATAC-seq, 4C, in utero electroporation (IUE), H3K4me3-PLAC-seq, western blot, quantification, and statistical analysis were performed according to published protocols. Information for the mouse lines used and generated, detailed experimental procedures, and analyses can be found in *SI Appendix*.

## Supplementary Material

Appendix 01 (PDF)

## Data Availability

ChIP-seq, CUT&RUN, and ATAC-seq generated in this study have been deposited in the Gene Expression Omnibus (GEO) under the accession number GSE254693 (28).
