# Supplementary material for: A conserved molecular logic for neurogenesis to gliogenesis switch in the cerebral cortex
Source: Proc Natl Acad Sci U S A. 2024 May 7;121(20):e2321711121. doi: 10.1073/pnas.2321711121 (PMC11098099; doi:10.1073/pnas.2321711121)
Supplement: Supplementary file 1 — Appendix 01 (PDF) [file pnas.2321711121.sapp.pdf]

Supporting Information for

A conserved molecular logic for neurogenesis to gliogenesis switch in the cerebral cortex

Xiaoyi G. Liang[1], Kendy Hoang[1], Brandon L. Meyerink[2,3], Pratiksha Kc[2], Kitt Paraiso[4], Li Wang[5, 6], Ian R. Jones[7], Yue Zhang[1], Sol Katzman[8], Thomas S. Finn[1], Jeremiah Tsyporin[1], Fangyuan Qu[1], Zhaoxu Chen[1], Axel Visel[4, 9, 10], Arnold Kriegstein[5,6], Yin Shen[6,7], Louis-Jan Pilaz[2,11], and Bin Chen[1,*]

Bin Chen

Email: bchen@ucsc.edu

This PDF file includes:

Supporting text

Figures S1 to S8

SI References

Supporting Information Text

Online Materials and Methods

Ethics statement

Deidentified tissue samples were collected with prior informed consent in strict observance of legal and institutional ethical regulations. All protocols were approved by the Human Gamete, Embryo, and Stem Cell Research Committee (GESCR) and Institutional Review Board (IRB) at the University of California, San Francisco.

Animals

Experiments were performed according to protocols approved by the Institutional Animal Care and Use Committee at University of California at Santa Cruz, University of South Dakota Sanford School of Medicine, and Lawrence Berkeley National Laboratory.

The day of the vaginal plug detection was designated as E0.5. The day of birth was designated as P0. The sexes of the embryonic and early postnatal mice were not determined.

The generation and genotyping of the $Smo^{fl/fl}$ (JAX no. 004526) (1), $Rosa26^{SmoM2/+}$ (JAX no. 005130) (2), $Gli3^{fl/fl}$ (JAX no. 008873) (3), $Pax6^{fl/fl}$ (JAX no. 028032) (4), $Emx1^{Cre/+}$ (JAX no. 005628) (5), $Olig2^{+/-}$ mice (JAX no. 025567) (6), and *hGFAP-Cre* (JAX no. 004600) (7) mice were described previously.

Generation of the $Olig2^{\Delta e14414/+}$ mice: These mice were generated using CRISPR/Cas9 method. Two sgRNAs (5' Guide #1 TCATCTGCGACCTAACAGAC TGG and 3' Guide #2 GTTCCCACGGTTGCTAAGGA AGG) were chosen and Cas9 protein/sgRNA complexes were injected into zygotes of C57BL/6N mice. 174 zygotes were transferred into surrogate female mice and 17 pups were born. The mice were genotyped using 4 primers: wt-F2 (ATGTGGCAAAGCTAGAGGAGATGCC) and wt-R2 (CAAGACTCCGGTGTGAAAGCTCCAG) for the wild type allele (product: 792 bp), mut-F2 (GATGGCCGACAGCACAGGAAGTATT) and wt-R2 for the mutant allele (wild type product: 5337 bp; mut allele: ~610 bp). The PCR products were sequenced to confirm genotyping results. Four male founders were obtained. Each founder mouse was bred to female C57BL/6N mice to obtain F1 mice. Progenies from F1 mice were bred to mice from the same founder to generate homozygous mutants, and bred to $Olig2^{+/-}$ mice to generate trans-heterozygous mice. Mice from all 4 founders showed the same phenotype.

Generation of $Olig2^{\Delta e14415/+}$, $Olig2^{\Delta GBS2/+}$, and $Olig2^{\Delta e14416/+}$ mice: These mice were generated using the iGONAD method (8). Two guide RNAs and a HDR donor oligo as repair template were designed for each enhancer. The HDR donor oligos contain an EcoRI restriction site for easy genotyping. Each founder mouse was bred to female C57BL/6N mice to obtain F1 mice. Progenies from F1 mice were bred to mice from the same founder to generate homozygous mutants, and bred to $Olig2^{+/-}$ mice to generate trans-heterozygous mice. Mice from all founders for the same enhancer showed the same phenotype.

The sequences for the gRNAs and HDR oligos, and genotyping primers are:

GBS2 (chr16: 91191215-91194007)
5' guide: TAATCCAGAAATACCATGTTTGG
3' guide: GACCTTTAAACATGAGAGACTGG

HDR oligo:
TAAGTACTACACCAGCGAAGCTGTCTCTCTAGCCCCAAACGAATTCCTCATGTTTAAAGGTC
ACAGAAATGAACTCCCCGAGAGGC
Genotyping primers:
Outer primers
Forward primer (e2-FW): CTTGAGACGTGCAGGGTAGG
Reverse primer (e2-out-RV): CTCCTGTTTCAGCCTCTCGG
Wild-type product length: 3401 bp
Product length w/ recombination: 456 bp; after EcoRI digestion: 402 bp + 54 bp
Inner primers (Forward same as outer)
Forward primer (e2-FW): CTTGAGACGTGCAGGGTAGG
Reverse primer (e2-in-RV): GGGGATGAGCCATAAACTGGG
Product length: 561 bp

e14415 (chr16:91173251-91175250)
5' guide: GAGAAACGCTCCATCTCTAAGGG
3' guide: CCCCTAATATGTACACACATAGG
HDR oligo:
TGCAAGCGCATGCAAGCCTGTGTCTCTGACGATACCCTTAGAATTCGTGTACATATTAGGGG
CAGGGTTAAATATATGGCATAACC
Genotyping primers
Outer primers
Forward primer (e3-FW): GCCTGCCTGTTTAGAGGTGT
Reverse primer (e3-out-RV): TCTGCAAAGTGAACGCAAGC
Wild-type product length: 2529 bp
Product length w/ recombination: 367 bp; after EcoRI digestion: 110 bp + 257 bp
Inner primers (Forward same as outer)
Forward primer (e3-FW): GCCTGCCTGTTTAGAGGTGT
Reverse primer (e3-in-RV): CCATTCAGCCAGCATTCAGC
Product length: 411 bp

GBS3/E14416 (chr16:91300728-91304721)
5' guide: TCTCTGGGAGTAAAGACATCAGG
3' guide: AGCACACTTCCCATTTAACTTGG
HDR oligo:
AGAGAGAGAGAGTATAGGGGCTGATCTCTGGGAGTAAAGAGAATTCAAATGGGAAGTGTGC
TCTGCTTAGAAAGGGAAGGTGTGTG
Genotyping primers:
Outer primers
Forward primer (e1-FW): TCAGTCCTTATCTCCCGCCT
Reverse primer (e1-out-RV): GGAGTGGCTTCTTCCCAACA
Wild-type product length: 4592 bp
Product length w/ recombination: 672 bp; after EcoRI digestion: 211 bp + 461 bp
Inner primers (Forward same as outer)
Forward primer (e1-FW): TCAGTCCTTATCTCCCGCCT
Reverse primer (e1-in-RV): GGCCAGACCAAACCTGGATTA
Product length: 587 bp

The enhancer reporter mice were generated and analyzed as described previously (9-11). The enhancer sequences were amplified by PCR from human or mouse genomic DNA where applicable, cloned into the vector plasmid *pPCR4-Shh::lacZ-11* (Addgene #139098) (10, 11), and sequenced by Miseq. After pronuclear microinjection, the F0 embryos were harvested at E11.5 or E16.5 and stained with X-gal to detect β-galactosidase (LacZ) activity. The embryos were

genotyped by PCR and Sanger sequencing. All the enhancer data have been uploaded into the Vista Enhancer Browser.

Immunohistochemistry

4% paraformaldehyde was delivered to mice via trans-cardiac perfusion. Brains were post-fixed in 4% paraformaldehyde, 0.1% saponin, and PBS for 24 hours at 4°C, followed by cryoprotection in 30% sucrose in PBS. Brains were sectioned via cryostat at 12 μm or 20 μm.

Immunohistochemistry was performed using standard protocols. Briefly, brain sections were permeabilized with 0.3% Triton X- 100 in phosphate buffered saline (PBS) for 10 min. Slides were immersed in citrate acid buffer (10mM citric acid monohydrate, 0.5% Tween-20, pH 6.0), and brought into a steamer for 25 min. Slides were cooled down to RT after steaming and then incubated in a blocking buffer (5% Horse serum, 0.3% Triton X-100 in PBS) for 30 minutes. Blocking buffer was removed, and the sections were incubated with primary antibodies (diluted in the blocking buffer) for overnight at 4°C.

The following primary antibodies were used in this study: OLIG2 (Mouse, Millipore 3319819), OLIG1 (Rabbit, Abcam ab124908), GLI3 (Goat, R&D Systems AF3690), SOX2 (mouse, BD Biosciences 561506), HOPX (Rabbit, Proteintech 11419-1-AP), PU.1 (Rabbit, Cell Signaling Technology #81886), SOX9 (Rabbit, Abcam ab185230), SOX10 (R&D System AF2864), PDGFRa (Rat, BD Pharmingen 558774), SP8 (Goat, SCBT Sc-104661), GSX2 (Rabbit, Millipore ABN162), ALDH1L1(Rabbit, Abcam ab87117), EGFR(Goat, R&D Systems AF1280), MAPK (Rabbit, Cell Signaling Technology #9101), ID1 (Rabbit, Biocheck, Inc BCH-1/#37-2), ID3 (Rabbit, Biocheck, Inc BCH-4/#17-3), KI67 (Mouse, BD Pharmingen #556003), ASCL1(Rabbit, Cosmo Bio SK-T01-003), GFP (Chicken, Aves Labs GFP-879484), Activated caspase 3 (Rabbit, Cell Signaling Technology #9661), H3k27me3 (Rabbit, Abcam ab190631), H3k4me3 (Rabbit, Abcam ab8580), and H3K27ac (Rabbit, Abcam ab4729).

The sections were washed in PBS, and incubated with secondary antibodies conjugated to Alexa 488, Alexa 546, Alexa 555, or Alexa 647 for 1.5 hour at room temperature. Secondary antibodies were from Jackson ImmunoResearch and Invitrogen. Finally, the sections were counterstained with DAPI for 5 mins before being mounted in Fluoromount-G (Invitrogen, 00495802).

EdU labeling

Timed pregnant mice were injected with a single dose of EdU (40 mg/kg body weight; Thermo Fisher Scientific, E10187) at E17.5. Brains were collected 2 hours after injection. EdU was detected via a click-chemistry reaction containing the following reagents per 1 ml of reaction: 950 μl 100 mM Tris PH 7.4, 40 μl 100 mM $CuSO_4$, 10 μl 200 mg/mL sodium ascorbate, and 1 μl Azide 555 (Invitrogen, A20012).

CUT&RUN-seq

The E16 wild type and *hGFAP-Cre; Rosa26*$^{SmoM2/+}$ cortices (n=3 each group) were dissected, and cells were dissociated with the Accumax (Sigma-Aldrich A7089) following the manufacturer's instructions. CUT&RUN was performed according to the published protocol (12) (n=3 biological replicates/antibody/genotype). H3K27ac, H3K4me3 and H3K27me3 antibodies were used in this study. 10,000 cells were used for each experiment, and 3 biological replicates were performed for each antibody and genotype. DNA libraries were constructed using NEBNext Ultra II DNA Library Prep Kit for Illumina (NEB #E7645, E7103) with index primers (NEB, E7645-S). Pippen size selection was used to remove the primer dimers. The libraries were sequenced via NextSeq 75 cycles High (PE37) platform with 1% PhiX control.

ChIP-seq

E15 wild type cortices were dissected. The cells were dissociated, fixed for 10 min with 1% formaldehyde and neutralized with glycine. ChIP-seq experiments were performed according to the published protocol (13) (n=3 biological replicates/antibody). Briefly, the cells were lysed, and the chromatin was sheared into 100~300 bp fragments. Immunoprecipitation reactions were performed using the Gli3 antibody. Sequencing libraries were generated from the ChIP-ed DNA and input DNA for control using Ovation Ultralow System V2 kit according to the manufacturer's protocol. Paired end sequencing (PE100) was performed on Illumina Hiseq4000 platform.

ATAC-seq

The *pCAG-EGFP* plasmids alone or *pCAG-ShhN* along with *pCAG-EGFP* plasmids were electroporated into cortical ventricular zone of wild type embryos at E13.5. E16 cortices from the electroporated brains were dissected, and cells were dissociated using the Worthington Papain Dissociation Kit. Fluorescence activated cell sorting (FACS) was performed to enriched for the GFP^+ cells. 50,000 cells were used for each ATAC-seq experiment. The libraries were prepared by Nextera DNA Library Prep Kit (Illumina, catalog # FC-121-1030).

4C-seq

E16 and P0 wild type cortices, LGE/CGE tissues were dissected and used for 4C-seq according to a published protocol (14). *Olig1* and *Olig2* promoters were used as viewpoints. Primers were designed using Primer3. The restriction enzymes (REs) used and primers were described below. 4C libraries were generated with dual index NEB primers (E7780-S). The pooled library has low complexity and about 20% PhiX was spiked in, and we sequenced the library using NextSeq 150 cycles MID output (PE75). The following pipeline were used to analyze the data https://github.com/deLaatLab/pipe4C (14).

We performed three 4C experiments using the *Olig2* promoter as the viewpoint, and two 4C experiments using the *Olig1* promoter as the viewpoint. The restriction enzymes (REs), sequencing primer, reading primer, outward primer, and non-reading primer for the 4C experiments were:

Gene: *Olig2*
1st RE: Csp6I
2nd RE: DpnII
Sequencing primer: TATTGTCACTGTAGCGTCAG
Reading primer: TACACGACGCTCTTCCGATCTTATTGTCACTGTAGCGTCAG
Outward primer: CTCCAAAGTGGCTAGGATTT
Non-reading primer:
ACTGGAGTTCAGACGTGTGCTCTTCCGATCTCTCCAAAGTGGCTAGGATTT

Gene: *Olig2*
1st RE: DpnII
2nd RE: Csp6I
Sequencing primer: TATTGTCACTGTAGCGTCAG
Reading primer: TACACGACGCTCTTCCGATCTTATTGTCACTGTAGCGTCAG
Outward primer: CTCCAAAGTGGCTAGGATTT
Non-reading primer:
ACTGGAGTTCAGACGTGTGCTCTTCCGATCTCTCCAAAGTGGCTAGGATTT

Gene: *Olig2*

1st RE: Csp6I
2nd RE: DpnII
Sequencing primer: CACACACATACAAAGCTGAG
Reading primer: TACACGACGCTCTTCCGATCTTATTGTCACTGTAGCGTCAG
Outward primer: CGGACGAGTTTGCAGATT
Non-reading primer:
ACTGGAGTTCAGACGTGTGCTCTTCCGATCTCTCCAAAGTGGCTAGGATTT

Gene: *Olig1*
1st RE: Csp6I
2nd RE: DpnII
Sequencing primer: TGTCCTTGTAAAATGTGATC
Reading primer: TACACGACGCTCTTCCGATCTTGTCCTTGTAAAATGTGATC
Outward primer: GATAACCCTCTCTGGCAAC
Non-reading primer:
ACTGGAGTTCAGACGTGTGCTCTTCCGATCTGATAACCCTCTCTGGCAAC

Gene: *Olig1*
1st RE: NlaIII
2nd RE: DpnII
Sequencing primer: GGTTGGCTGTTATAAAAATGC
Reading primer: TACACGACGCTCTTCCGATCTTGTCCTTGTAAAATGTGATC
Outward primer: GATAACCCTCTCTGGCAAC
Non-reading primer:
ACTGGAGTTCAGACGTGTGCTCTTCCGATCTGATAACCCTCTCTGGCAAC

H3K4me3 PLAC-seq

The dissociated human fetal cortical cells from GW15 and GW22 human fetal brain tissue were stained with antibodies for SOX2, HOPX, OLIG2, and PU.1. Distinct cell populations were isolated via FACS: ventricular radial glia (vRG, $SOX2^{+}HOPX^{low}$), outer radial glia (oRG, $SOX2^{+}HOPX^{high}$), glial progenitors (IMP/OPC/oligodendrocytes, $OLIG2^{+}$), and microglia ($PU.1^{+}$). Following cell sorting, we performed H3K4me3 proximity ligation-assisted ChIP-seq (PLAC-seq) using sorted cells, and applied the Model-based Analysis of PLAC-seq (MAPS) pipeline to call significant H3K4me3-mediated chromatin interactions at a resolution of 2 kb, as described in detail previously (15).

Western blot analysis

P0 cortices were dissected in ice cold 1X PBS with protease inhibitor. The tissue was homogenized in RIPA buffer by manually triturating with a P1000 pipet tip before incubating on ice for 20 minutes. The cell homogenate was centrifuged at 14,000 g for 10 minutes at 4°C. The supernatant was removed, and proteins were denatured at 100°C for 5 minutes in laemmli buffer. The samples were then run on an 8% SDS-PAGE at 70V for 2 hours, transferred to a PVDF membrane (Sigma, IPVH85R) at 150 mA for 90 minutes. The blot was blocked for one hour in 1% non-fat milk in 1X Tris-Buffered Saline, 0.1% Tween 20 (TBST). After blocking, GLI3 (Goat, R&D Systems AF3690, 1:500) and β-actin antibodies (Mouse, Abcam ab8226, 1:2000) were added and incubated at 4°C overnight. The blot was then washed with TBST and incubated in Donkey anti Goat secondary antibody (Abcam ab6885) and Donkey anti Mouse secondary antibody (Abcam ab205724) at a 1:20,000 dilution for one hour and images were processed using ImageStudioLite. Western blot analysis was done for 3 wild type and 3 *Smo cko* samples. Statistical significance was determined using the unpaired t-test. Significance was set as * for $p < 0.05$, ** for $p < 0.01$, *** $p < 0.001$ for and ****$p<0.0001$ all significance tests.

Image acquisition and analysis

Images for quantitative analyses were acquired with a Zeiss 880 confocal microscope. Laser power and gain were adjusted until <1% of pixels were saturated. Cell counting was performed on single z-slices with FIJI. Individual channels were adjusted with auto threshold "Moments," or a manual threshold was applied to discern high versus low expressing cells. The dilate, erode, and watershed functions were sequentially used before particles were analyzed with a circularity of 0.3-1.0 and size exclusion of >1µm. Brightfield images were acquired with a Zeiss AxioImager Z2 widefield microscope with a Zeiss AxioCam 506 (color) camera.

Quantification of the Gli3, and Olig2 protein expression levels in the cortical VZ/SVZ of P0 wild type and *Smo cko* mouse brains: The brains were sectioned at 12 µm and Olig2/Gli3 immunostaining was performed on the section. 40X images were taken by using ZEISS 880 Confocal microscope. The individual cells in the VZ/SVZ were selected based on DAPI staining. The sum of the values of the pixels in the selection was calculated by FIJI as the integrated density. The average cell fluorescence value was calculated by dividing the integrated density by the area of selection. We selected 100-150 cells in the VZ/SVZ to calculate the average cell fluorescence value in each channel. The data were visualized by normalizing the max average cell fluorescence value to 1000 in each channel to show the differential expression level of the Gli3 and Olig2 proteins. The cell with a 1000 value is the brightest cell in the image. The log-linear regression analysis and Kolmogorov-Smirnov statistical tests were performed to compare the expression and distribution of Gli3 and Olig2 expression of the 2 sets of cells. Statistical analysis was performed using GraphPad Prism 5.0, or R. Only single Z-slice confocal images were used in cell quantifications. For each brain, the numbers of marker$^+$ cells in the cortex were quantified in 300- or 350-µm-wide regions from 3 sections. Care was taken to match the anterior-posterior, medial-lateral positions for the chosen areas between the mutant and control genotypes. For each genotype and each age, 3 different brains were analyzed. Data are shown as mean + SEM. Statistical significance for single comparisons was determined using the unpaired t-test. Significance was set as * for $p < 0.05$, ** for $p < 0.01$, *** $p < 0.001$ for and ****$p<0.0001$ all significance tests.

Supporting Figures

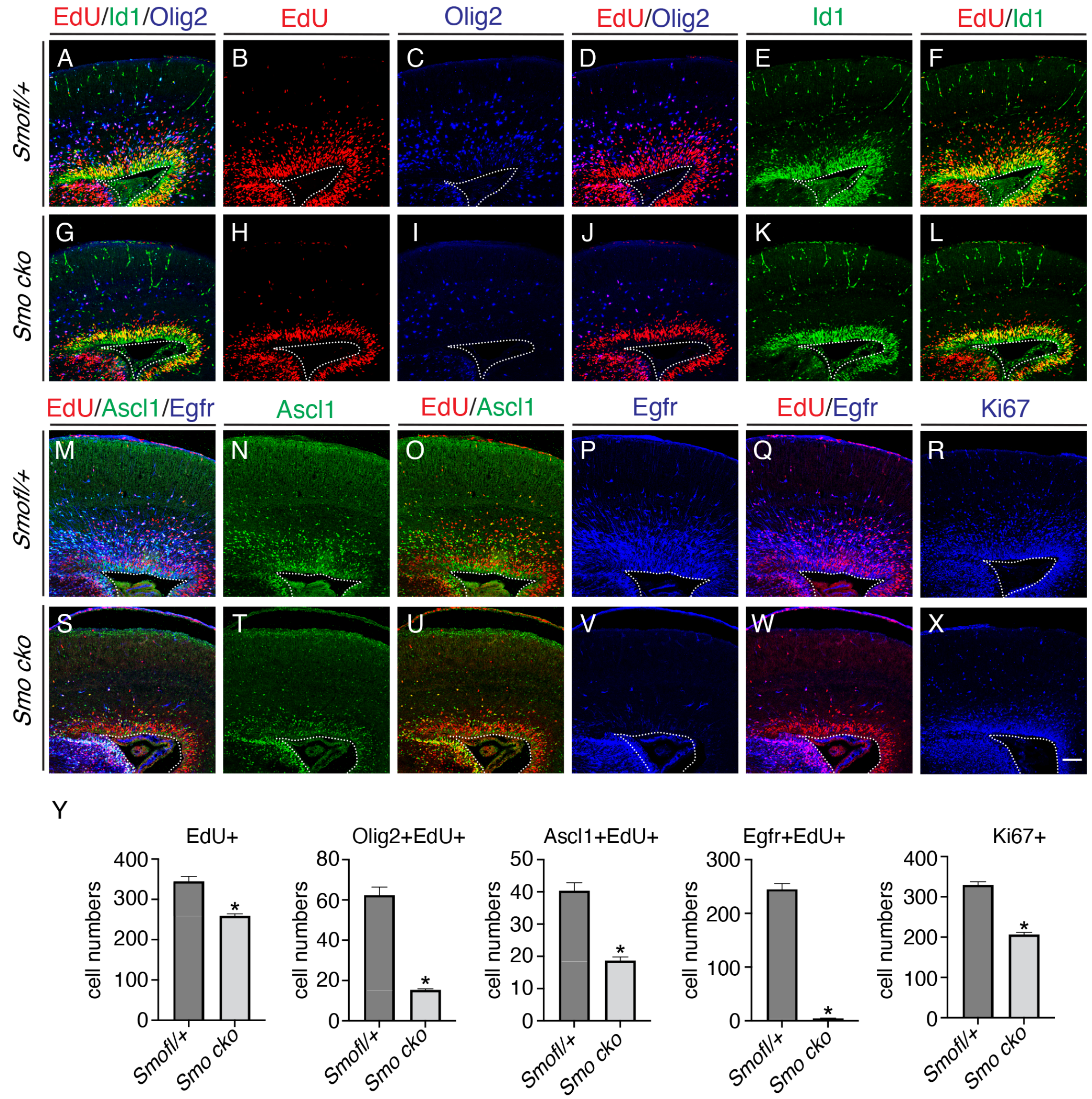

Fig. S1. Fewer MIPCs proliferate in the VZ/SVZ of E17.5 *Smo cko* mouse cortices. A-L, EdU labeling and expression of Olig2 and Id1 in the *Smo*$^{fl/+}$ (A-F) and *hGFAP-Cre; Smo*$^{fl/fl}$ (*Smo cko*) (G-L) cortices. EdU was injected 2 hours before brain collection. M-Q and S-W, EdU labeling and expression of Ascl1 and Egfr in the *Smo*$^{fl/+}$ (M-Q) and *Smo cko* (S-W) cortices. R and X, Immunostaining using Ki67 antibody of the *Smo*$^{fl/+}$ (R) and *Smo cko* (X) brain sections. Images were taken at the rostral-middle position along the rostral-caudal axis. Y, Quantifications of the EdU^{+}, Olig2^{+}EdU^{+}, Ascl1^{+}EdU^{+}, Egfr^{+}EdU^{+} and Ki67^{+} cells per section in the VZ/SVZ of *Smo*$^{fl/+}$ and *Smo cko* cortices. Numbers represent means + SEM (n=3 mice per genotype). *, $P<0.05$; unpaired Student's t test. Scale bars: 100 μm in X, applies to A-X.

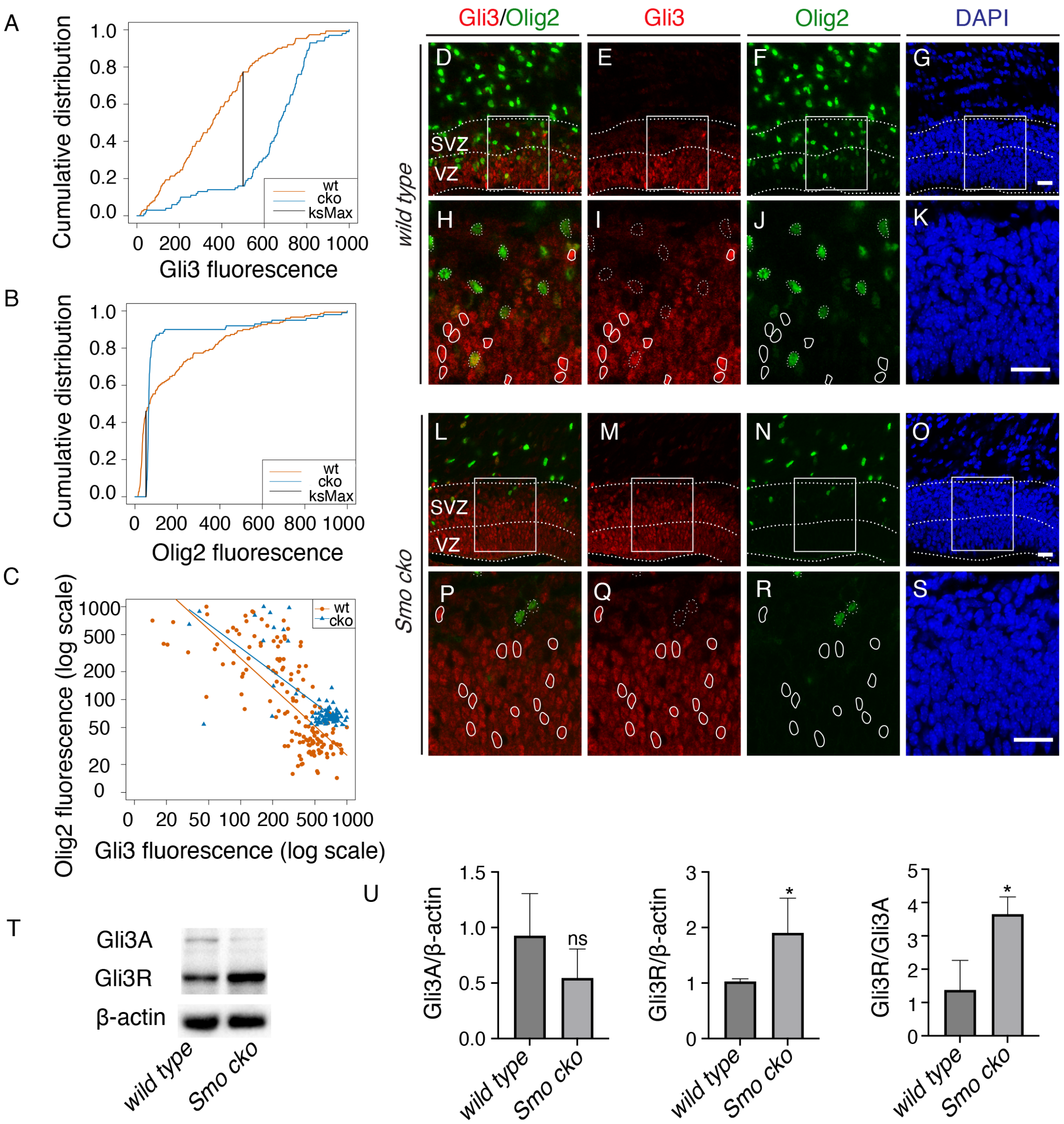

Fig. S2. Negative correlation between Gli3 and Olig2 expression in P0 cortical progenitor cells. A and B, Empirical cumulative distribution functions of wild type (wt) and *Smo cko* (cko) for Gli3 (A) and Olig2 (B) fluorescence signal. Applying the Kolmogorov-Smirnov 2-sample test to determine if the distributions are different yields two-sided p-values of 2.2e-16 (A) and 1.9e-11 (B). C, Quantification of Gli3 and Olig2 protein expression in wild type (orange) and *Smo cko* (blue) cortical progenitors. Each dot represents a cell. The cell with a 1000 value is the brightest cell in the field. A linear regression of the log-scaled values of Gli3 and Olig2 have negative slopes of -1.04 (wild type) and -0.85 (*Smo cko*), and Adjusted R-squared values of 55% (wild type) and 52% (*Smo cko*). D-S, Sample images of Gli3 and Olig2 immunostaining in wild type (D-K) and *Smo cko* (L-S) cortical progenitors used for quantification. H-K and P-S show the higher magnification images of the boxed areas in D-G and L-O, respectively. Images were taken at the rostral-middle position along the rostral-caudal axis. T and U, Western blot (T) and quantification (U) (n=3 replicates/genotype) of Gli3R, Gli3A protein levels and comparison of Gli3R/Gli3A ratios in P0 wild type and *Smo cko*

cortices. Scale bars: 20 μm in G, applies to D-G; 20 μm in K, applies to H-K; 20 μm in O, applies to L-O; and 20 μm in S, applies to P-S.

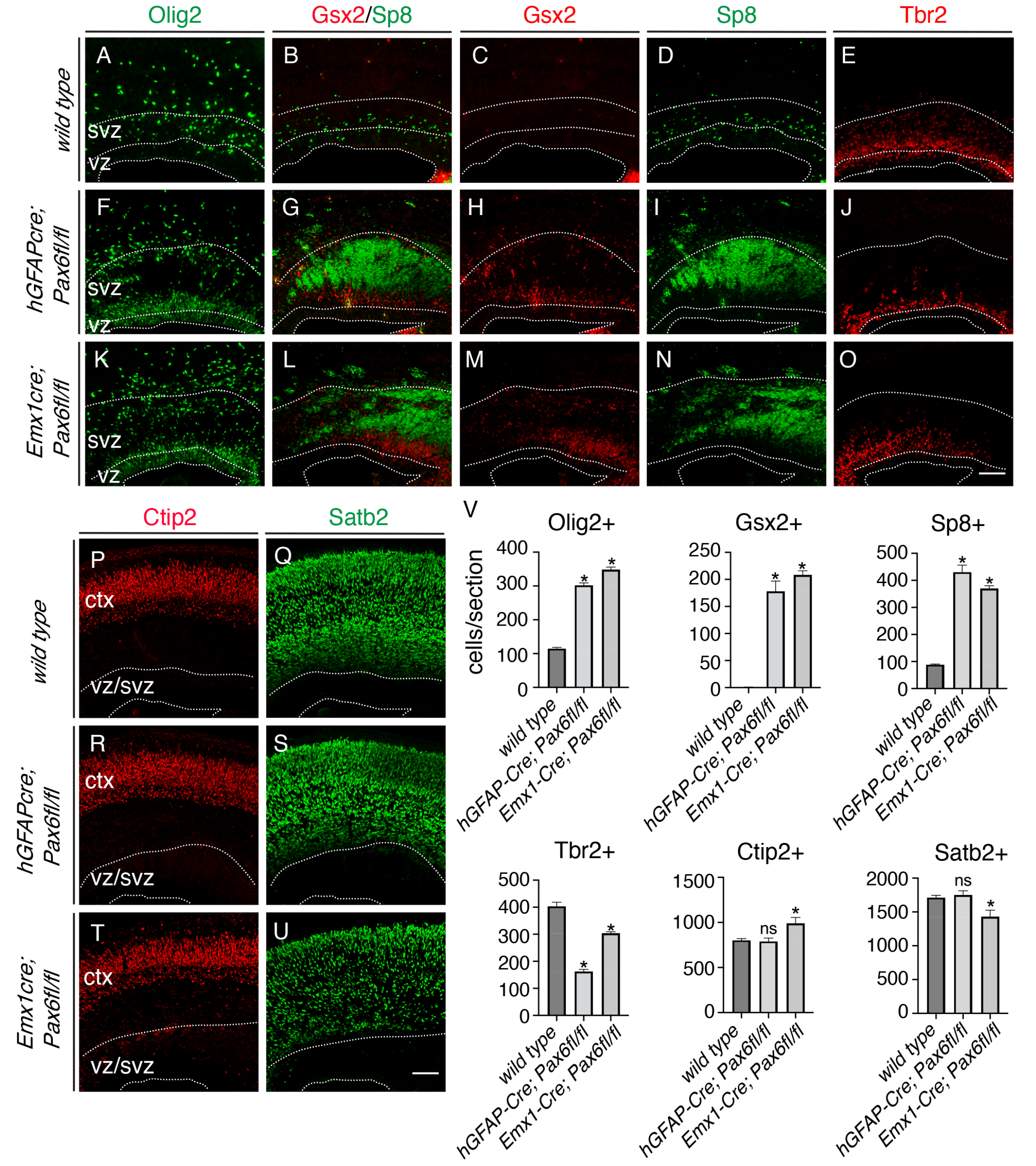

Fig. S3. Pax6 represses Olig2 expression and olfactory bulb interneuron lineage. A-U, Immunostaining of Olig2, Gsx2, Sp8, Tbr2, Ctip2 and Satb2 in E16.5 wild type (A-E, P-Q), *hGFAPcre; Pax6*$^{fl/fl}$ (F-J, R-S) and *Emx1-cre; Pax6*$^{fl/fl}$ (K-O, T-U) cortices. Images were taken at the rostral-middle position along the rostral-caudal axis. V, Quantification of Olig2^{+}, Gsx2^{+}, Sp8^{+} and Tbr2^{+} cells in the VZ/SVZ, Ctip2^{+} and Satb2^{+} cortical neurons per 350-μm wide section. Numbers represent means + SEM (n=3 mice per genotype). *, P<0.05; unpaired Student's t test. Scale bar: 100 μm in O, applies to A-O; 100 μm in U, applies to P-U.

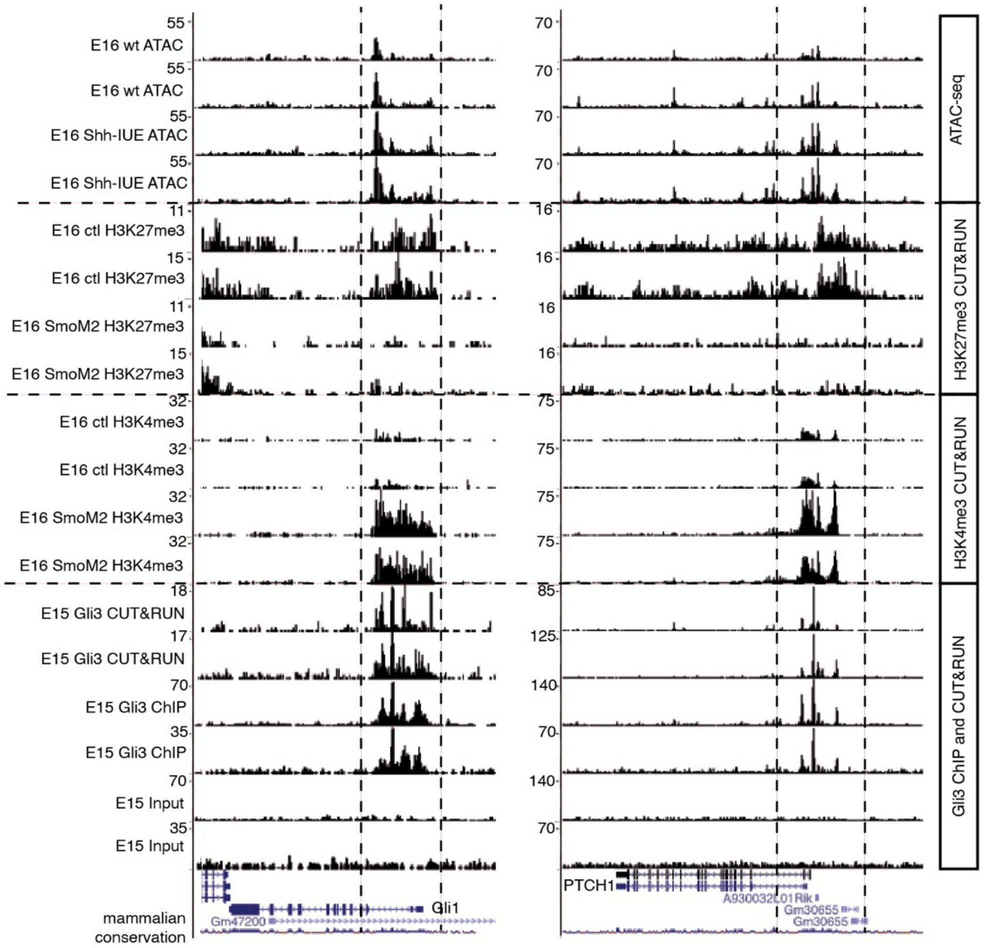

Fig. S4. Establishing ChIP-seq, CUT&RUN, and ATAC-seq methods. ChIP-seq and CUT&RUN show Gli3 binds to the promoters of both *Gli1* and *Ptch1* genes. In the *hGFAP-Cre; Rosa26SmoM2* (SmoM2) cortices, promoters of *Gli1* and *Ptch1* show increased H3K4me3 and less H3K27me3. The *pCAG-EGFP* plasmids or *pCAG-EGFP* and *pCAG-ShhN* plasmids were electroporated into the VZ of E16 wild type embryos, and GFP^{+} cells were enriched by FACS at P0 and used for ATAC-seq. ATAC-seq showed increased accessibility of Gli1 and Ptch1 promoters when *pCAG-ShhN* was electroporated. Tracks for 2 biological replicates for each experiment were shown.

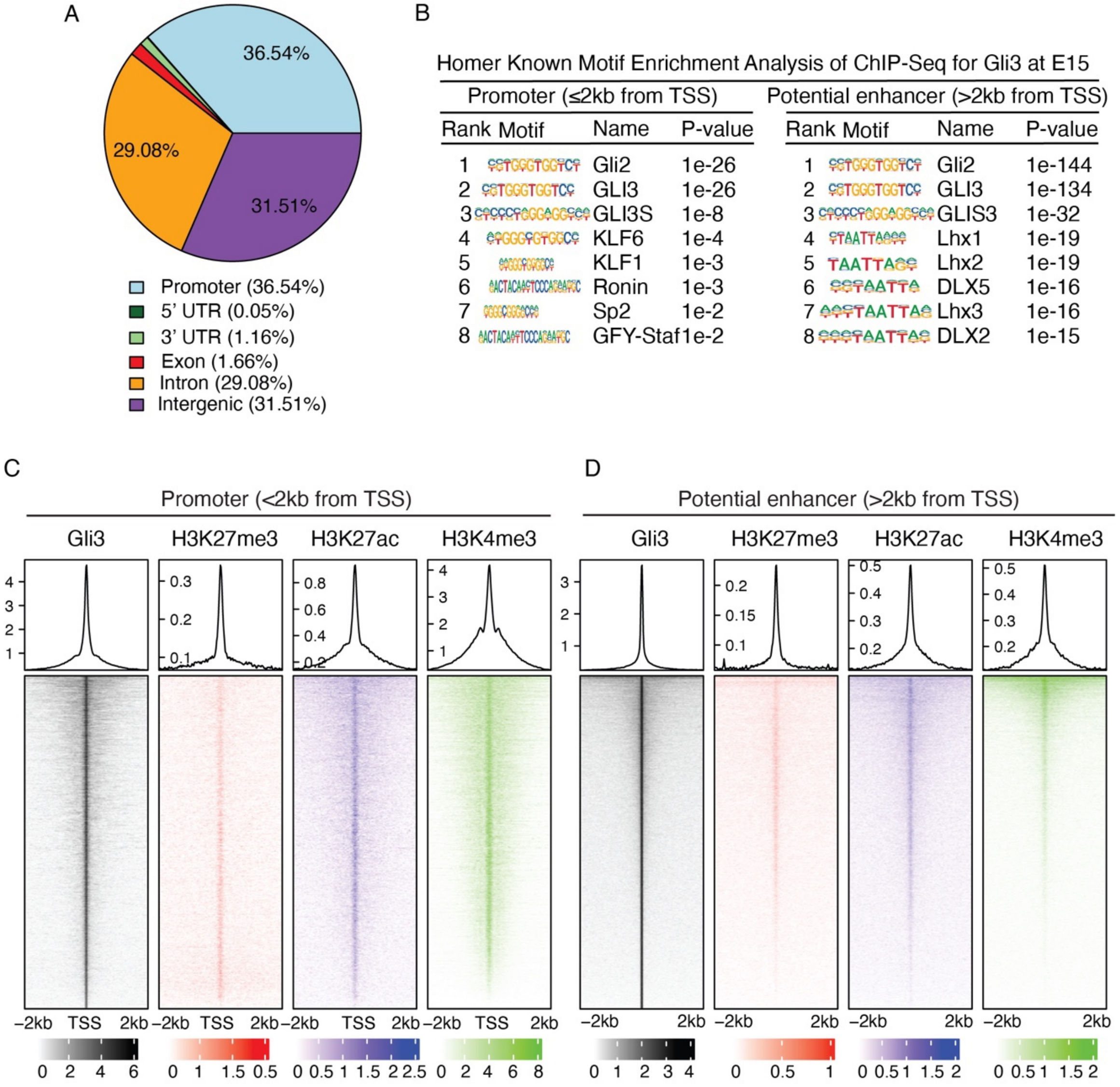

Fig. S5. ChIP-seq experiments reveal Gli3 binds to both promoter and enhancer regions. A, A pie chart showing the distribution of the Gli3 binding sites in the genome of cortical cells. B, The most enriched known motifs for the Gli3 binding promoter and potential enhancer regions. C and D, Heat map showing Gli3 binding sites in the promoter (C) and enhancer (D) regions are enriched in H3K27me3, H3K27ac, and H3K4me3.

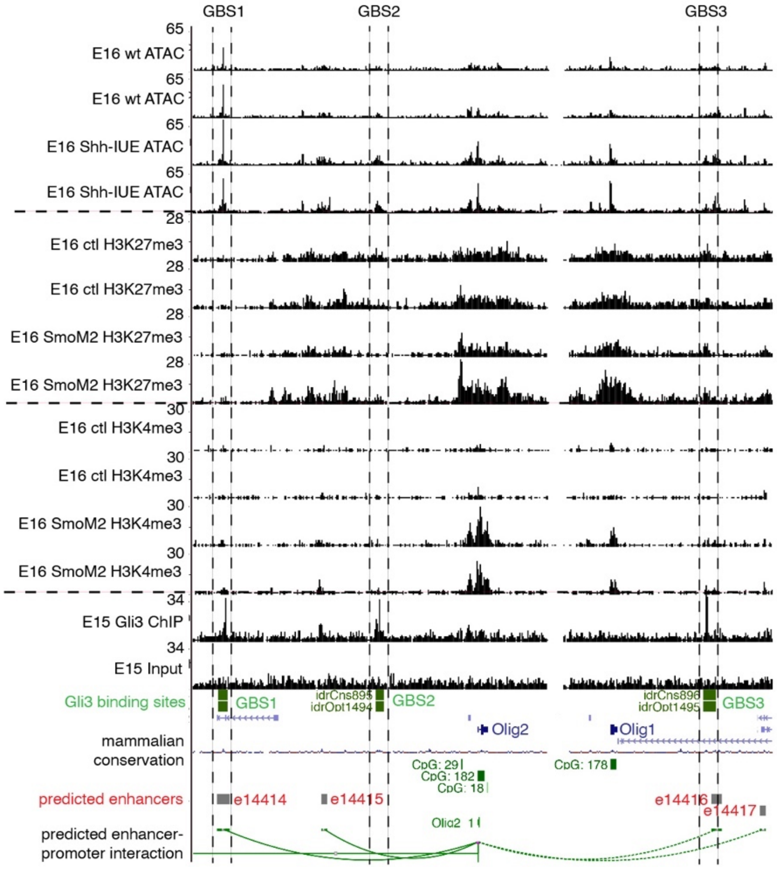

Fig. S6. Gli3 binds to 3 different sites (GBS1, GBS2, and GBS3) at the *Olig1/2* loci. GBS1 overlaps with predicted enhancer e14414, and GBS3 overlaps with e14416. ATAC-seq shows increased accessibility at the 3 GBS sites when Shh was overexpressed in the cortex. Wavy green lines represent predicted interactions between enhancers and the *Olig2* promoter.

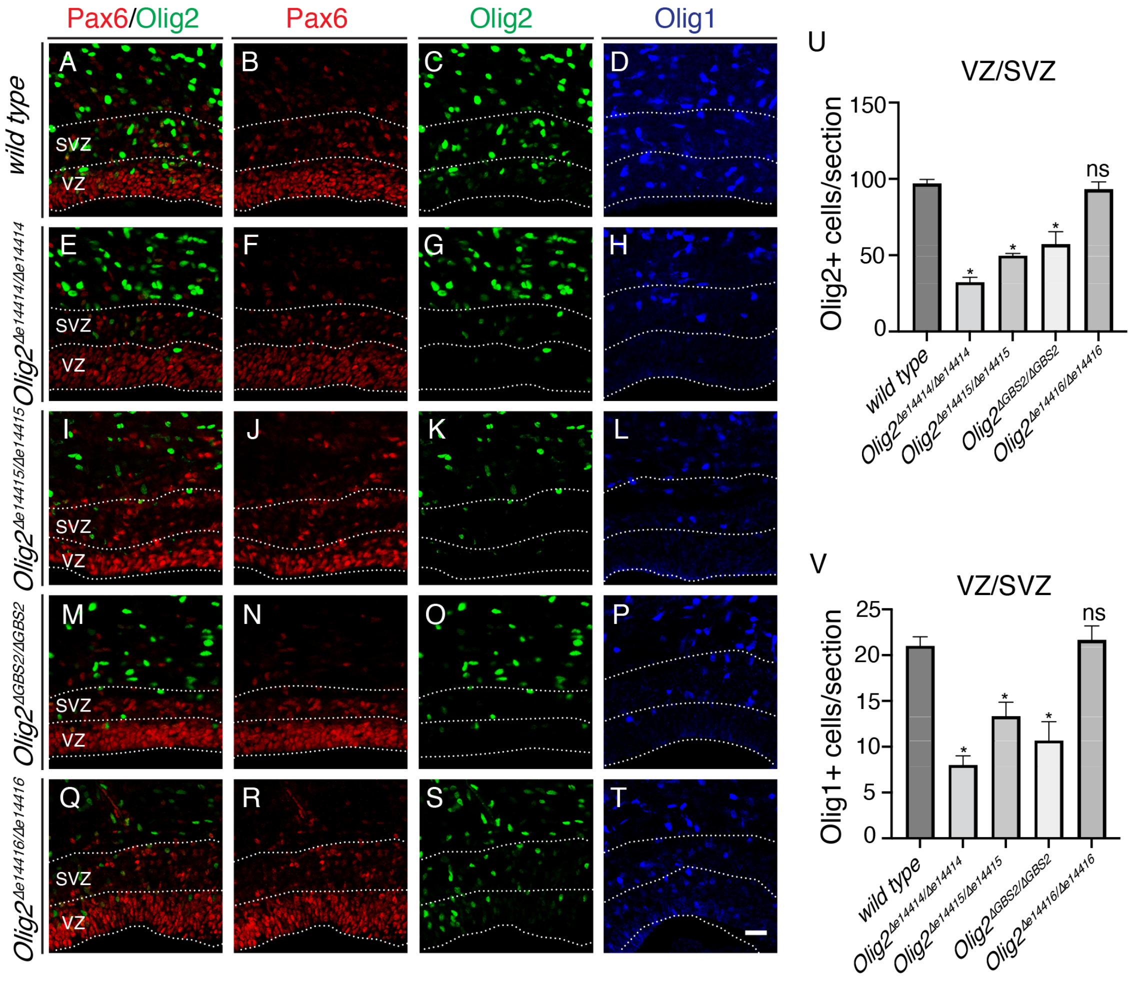

Fig. S7. Deletion of enhancer e14414, e14415 or GBS2 reduces Olig2 and Olig1 expression in cortical VZ/SVZ at P0. A-T, Immunostaining of Pax6, Olig2, Olig1 in the cortices of wildtype, *Olig2*$^{\Delta e14414/\Delta e14414}$, *Olig2*$^{\Delta e14415/\Delta e14415}$, *Olig2*$^{\Delta GBS2/\Delta GBS2}$ and *Olig2*$^{\Delta e14416/\Delta e14416}$ mice. Pax6 staining delineated the VZ and SVZ. Images were taken at the rostral-middle position along the rostral-caudal axis. U and V, Quantification of Olig2$^+$ and Olig1$^+$ cells per 350-μm wide section. Numbers represent means + SEM (n=3 mice per genotype). *, P<0.05; unpaired Student's t test. Scale bar: 20 μm in T, applies to A-T.

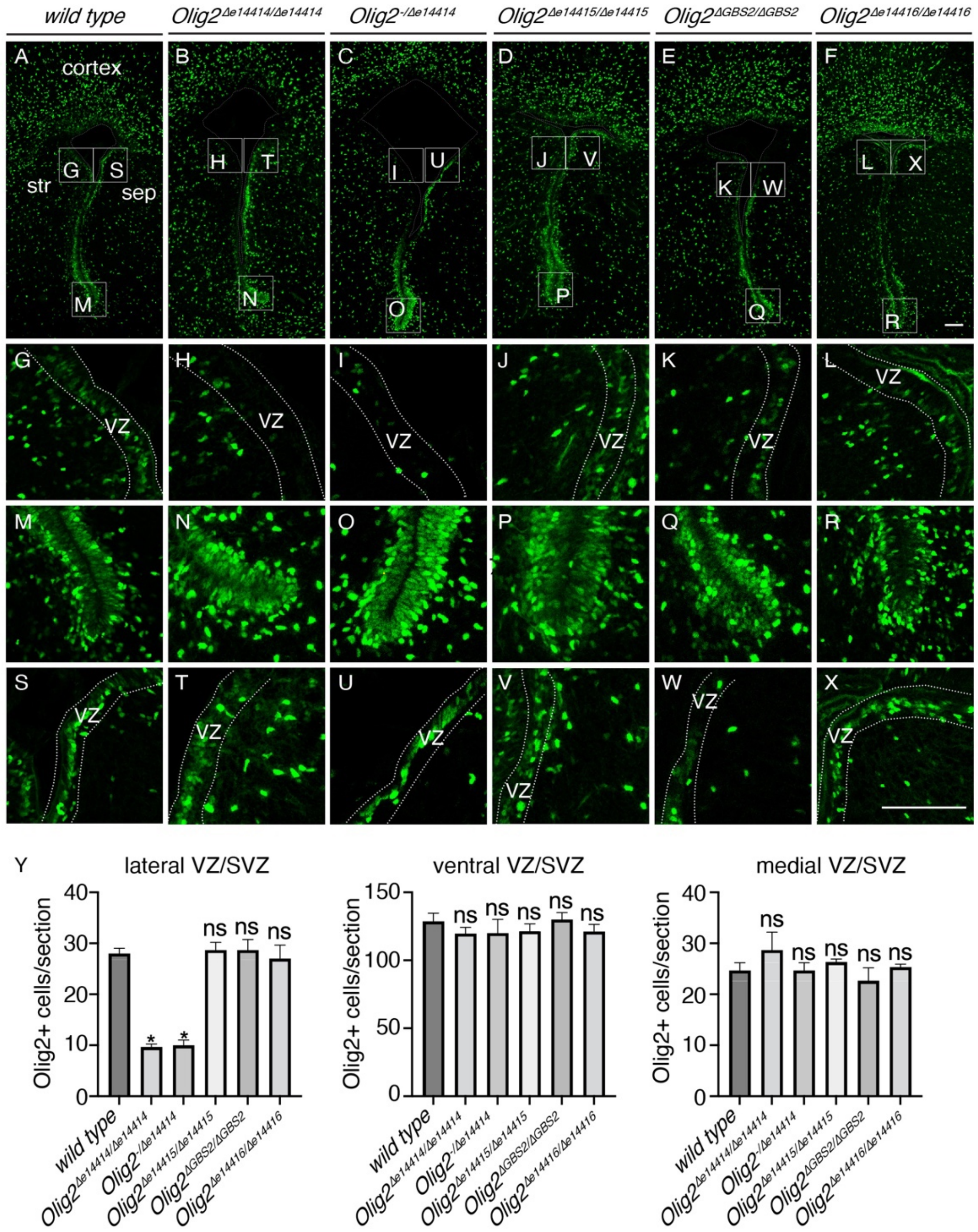

Fig. S8. Enhancer e14414 regulates *Olig2* expression in lateral VZ/SVZ of the forebrain. A-X, Immunostaining of Olig2 in the brains of P0 wild type (A, G, M and S), *Olig2*$^{\Delta e14414/\Delta e14414}$ (B, H, N and T), *Olig2*$^{-/\Delta e14414}$ (C, I, O and U), *Olig2*$^{\Delta e14415/\Delta e14415}$ (D, J, P and V), *Olig2*$^{\Delta GBS2/\Delta GBS2}$ (E, K, Q and W) and *Olig2*$^{\Delta e14416/\Delta e14416}$ (F, L, R and X) mice. G-X showing the high magnification images of the boxed areas in A-F. G-L showing Olig2 staining in the lateral VZ/SVZ. M-R showing Olig2 expression in the ventral VZ/SVZ. S-X showing Olig2 staining in the medial VZ/SVZ. Images were taken at the rostral-middle position along the rostral-caudal axis. Y, Quantifications of Olig2^{+} cells in lateral, ventral brain and medial VZ/SVZ. Numbers represent means + SEM (n=3 mice per genotype). *, $P<0.05$; unpaired Student's t test. Scale bar: 100 μm in F, applies to A-F; 100 μm in X, applies to G-X.

SI References

1. F. Long, X. M. Zhang, S. Karp, Y. Yang, A. P. McMahon, Genetic manipulation of hedgehog signaling in the endochondral skeleton reveals a direct role in the regulation of chondrocyte proliferation. *Development* **128**, 5099-5108 (2001).
2. J. Jeong, J. Mao, T. Tenzen, A. H. Kottmann, A. P. McMahon, Hedgehog signaling in the neural crest cells regulates the patterning and growth of facial primordia. *Genes Dev* **18**, 937-951 (2004).
3. S. Blaess, D. Stephen, A. L. Joyner, Gli3 coordinates three-dimensional patterning and growth of the tectum and cerebellum by integrating Shh and Fgf8 signaling. *Development* **135**, 2093-2103 (2008).
4. R. Ashery-Padan, T. Marquardt, X. Zhou, P. Gruss, Pax6 activity in the lens primordium is required for lens formation and for correct placement of a single retina in the eye. *Genes Dev* **14**, 2701-2711 (2000).
5. J. A. Gorski *et al.*, Cortical excitatory neurons and glia, but not GABAergic neurons, are produced in the Emx1-expressing lineage. *J Neurosci* **22**, 6309-6314 (2002).
6. M. Zawadzka *et al.*, CNS-resident glial progenitor/stem cells produce Schwann cells as well as oligodendrocytes during repair of CNS demyelination. *Cell stem cell* **6**, 578-590 (2010).
7. L. Zhuo *et al.*, hGFAP-cre transgenic mice for manipulation of glial and neuronal function in vivo. *Genesis* **31**, 85-94 (2001).
8. Y. Imai, A. Tanave, M. Matsuyama, T. Koide, Efficient genome editing in wild strains of mice using the i-GONAD method. *Sci Rep* **12**, 13821 (2022).
9. A. Visel, S. Minovitsky, I. Dubchak, L. A. Pennacchio, VISTA Enhancer Browser--a database of tissue-specific human enhancers. *Nucleic Acids Res* **35**, D88-92 (2007).
10. E. Z. Kvon *et al.*, Comprehensive In Vivo Interrogation Reveals Phenotypic Impact of Human Enhancer Variants. *Cell* **180**, 1262-1271 e1215 (2020).
11. M. Osterwalder *et al.*, Characterization of Mammalian In Vivo Enhancers Using Mouse Transgenesis and CRISPR Genome Editing. *Methods Mol Biol* **2403**, 147-186 (2022).
12. P. J. Skene, S. Henikoff, An efficient targeted nuclease strategy for high-resolution mapping of DNA binding sites. *eLife* **6** (2017).
13. W. L. McKenna *et al.*, Mutual regulation between Satb2 and Fezf2 promotes subcerebral projection neuron identity in the developing cerebral cortex. *Proc Natl Acad Sci U S A* **112**, 11702-11707 (2015).
14. P. H. L. Krijger, G. Geeven, V. Bianchi, C. R. E. Hilvering, W. de Laat, 4C-seq from beginning to end: A detailed protocol for sample preparation and data analysis. *Methods* **170**, 17-32 (2020).
15. M. Song *et al.*, Cell-type-specific 3D epigenomes in the developing human cortex. *Nature* **587**, 644-649 (2020).
